# Optimal allocation of antenatal and young child nutrition interventions: an individual-based global burden of disease calibrated microsimulation

**DOI:** 10.1186/s44263-024-00120-y

**Published:** 2025-01-15

**Authors:** Alison Bowman, Sylvia Lutze, James Albright, Nathaniel Blair-Stahn, Hussain Jafari, Simar Kaur, Caroline Kinuthia, Rajan Mudambi, Patrick Nast, Alix Pletcher, Abraham Flaxman

**Affiliations:** 1 Institute for Health Metrics and Evaluation, University of Washington, Seattle, WA USA; 2 Department of Health Metrics Sciences, University of Washington, Seattle, WA USA

**Keywords:** Maternal and child health, Simulation model, Health policy, Optimal allocation, Antenatal care, Young child feeding, Child nutrition

## Abstract

**Background:**

Undernutrition remains a global crisis and is a focus of Sustainable Development Goals. While there are multiple known, effective interventions, complex interactions between prevention and treatment and resource constraints can lead to difficulties in allocating funding. Simulation studies that use in silico simulation can help illuminate the interactions between interventions and provide insight into the cost-effectiveness of alternative packages of options.

**Methods:**

We developed an individual-based microsimulation model based on the Global Burden of Disease (GBD) 2021 study data to test a range of nutrition interventions, including antenatal interventions (iron and folic acid, multiple micronutrients, and balanced energy protein supplementation) and child interventions (treatment for severe acute malnutrition, treatment for moderate acute malnutrition, and wasting prevention with small-quantity lipid-based nutrient supplements). We also developed an analytic approach to process the results of the microsimulation and identify the optimal intervention funding allocation for a given budget size. We use Ethiopia as an example in this paper.

**Results:**

In our illustrative example of Ethiopia, the reallocation of the baseline budget to minimize disability-adjusted life years (DALYs) resulted in first funding the antenatal multiple micronutrients to their maximum coverage and then funding treatment for severe child acute malnutrition. Relative to the baseline allocation, the reallocation optimized to minimizing DALYs resulted in 592,000 fewer annual DALYs, constituting an 8.3% reduction in total DALYs in Ethiopia.

For budgets larger than the baseline, our model recommended funding first targeted moderate acute malnutrition treatment, second universal moderate acute malnutrition treatment, third wasting prevention with small-quantity lipid-based nutrient supplements, and fourth balanced energy protein supplementation.

**Conclusions:**

Our simulation is a novel model for estimating optimal allocation of spending on antenatal and child health nutrition interventions which accounts for the interaction between preventive and therapeutic approaches. Our illustrative results show that an optimized reallocation of current spending can substantially improve pregnancy-related and child health without additional funding. We hope this model can add validity and confidence to prior results to aid stakeholders in funding decisions.

**Supplementary Information:**

The online version contains supplementary material available at 10.1186/s44263-024-00120-y.

## Background

Undernutrition remains a global crisis, contributing significantly to the burden of disease and hindering progress toward the Sustainable Development Goal (SDG) to end hunger [1]. This persistent challenge demands urgent action and investment in proven interventions to address undernutrition during pregnancy and childhood.

Despite a range of known effective interventions, coverage remains insufficient and unevenly distributed, particularly in regions with the greatest need [2]. Two key areas where current practices lag are antenatal supplementation and the prevention and treatment of childhood acute malnutrition (AM). While iron and folic acid (IFA) supplementation is the standard of care in many settings, the World Health Organization (WHO) advocates for research on IFA-containing multiple micronutrient (MMN) supplements [3] and balanced energy and protein (BEP) supplementation for undernourished pregnant people [4]. Notably, there is suggestion that BEP supplementation targeted to undernourished pregnancies (in contrast to undernourished populations) may be a cost-effective strategy [5]. Furthermore, WHO recently issued new guidance on the prevention and treatment of AM among children under 5 [6]. These guidelines provide recommendations for the treatment of moderate acute malnutrition (MAM) in a targeted fashion in addition to the prior recommendations for treating severe acute malnutrition (SAM). It additionally issues new recommendations for the prevention of AM with strategies, including the consideration of medium- or small-quantity lipid-based nutrient supplementation (MQ-LNS or SQ-LNS), particularly in contexts of high food insecurity.

Even with the recognized need for scaling up nutrition interventions, resource constraints and the complexity of maximizing health impact with limited budgets pose significant challenges [7]. While prevention is generally more effective [8], resource allocation decisions must consider various factors, including intervention costs, population reach, and potential interactions between different interventions. In silico models offer a valuable tool for guiding decision-making by simulating intervention effectiveness and cost-effectiveness. Existing models like the multiple micronutrient supplementation (MMS) cost–benefit tool [9], Food Assistance Cost-Effectiveness Tool for Specialized Nutritious Foods (FACET4SNF) [10], community-based management of acute malnutrition (CMAM) costing tool [11], and World Breastfeeding Costing Initiative (WBCi) [12] provide insights into specific interventions, while the Micronutrient Intervention Modeling Project or MINIMOD focuses on micronutrient interventions [13]. The Optima Nutrition model [14], utilizing the Lives Saved Tool or LiST [15], offers a broader analysis but has limitations since MAM treatment cannot be run as an independent intervention from SAM treatment, limiting their ability to differentiate impact and interactions. The development of additional models that can incorporate targeted MAM treatment and evaluate optimal spending allocations could enhance decision-making by providing robust evidence across multiple platforms. In particular, conclusions that are robust across multiple models may reduce concerns regarding structural uncertainty [16].

In this interest, we developed an individual-based microsimulation model using Global Burden of Disease (GBD) 2021 study data to estimate the health impact of several nutrition-related interventions and paired it with an allocative efficiency analysis to determine allocation of intervention spending to optimize impact for a specified budget size(s). In this paper, we provide an overview of our simulation and include results specific to Ethiopia as an illustrative example.

Our team acknowledges that not all people who get pregnant or give birth are women, and we therefore strive to use more inclusive language that encompasses all identities without othering individuals. We are also aware that women often face unique challenges, and focused work on women and girls is essential to improving health for all. The accepted language in literature is often women-centric such as “maternal health” or “women of reproductive age.” Our team advocates for the use of language inclusive to all people that also honors the unique experiences and needs of women. Therefore, throughout this paper, we try to use gender inclusive language such as “pregnancy-related health” or “women and birthing people of reproductive age.” More information and a full glossary can be found in Additional file 1: Appendix 1.

## Methods

Our model consists of two main components: (1) an individual-based microsimulation model of health events and (2) an allocative efficiency analytic model that processes the results of the microsimulation model and outputs the optimal intervention funding allocation for a given budget size.

### Baseline health model

We utilize Vivarium [17], a mature, open-source, Python-based simulation framework for our baseline health model. Published examples of models utilizing Vivarium can be found elsewhere [5, 18, 19]. The data for this model is publicly available [20, 21]. For this application, our model consists of a closed cohort of simulated individuals that we track across discrete time steps. Simulated individuals are assigned various attributes (such as age, sex, risk factor exposures, disease status, and vital status) that evolve over time and influence their trajectory through the simulation. Specifically, at each time step, simulated individuals are subject to some probability of a disease event — for example, measles (noninfected individuals may become infected, and infected individuals may recover or die), which is modified by their other assigned attributes such as age, sex, and risk factor exposures. The model is calibrated such that individual-level heterogeneity reflects the appropriate magnitude across specific attributes, while averages across simulated individuals reflect appropriate population-level statistics.

The primary data source for our model was the GBD 2021 study, which estimates mortality and disability at the location-, year-, sex-, and age-specific level across hundreds of diseases, injuries, and risk factors [22–26]. Each time step spent affected by a morbidity-causing condition results in accumulation of years lived with disability (YLDs) in accordance with the disability weight (DW) of that condition utilized in the GBD study. Years of life lost (YLLs) are accumulated in accordance with the theoretical minimum risk life expectancy (TMRLE) specific to a simulated individual’s age at the moment of death as informed from the GBD study.

**Fig. 1:**
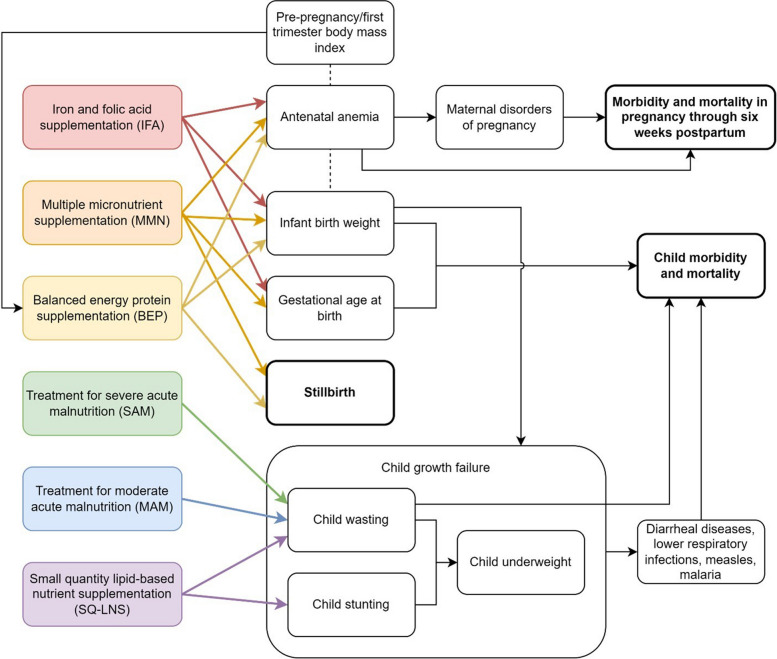
Diagram of all modeled components and the causal relationships between them in the microsimulation health model. Colored components represent interventions, and bolded components represent model outputs. Arrows represent causal impacts, and dashed lines represent noncausal correlations

Figure 1 represents all modeled components in our baseline health model and the interactions between them, which are discussed in more detail in the following sections. We separated our baseline health model into two distinct simulated population groups: pregnant women and birthing people and children under 5. Our simulation utilizes Monte Carlo methods to propagate parameter uncertainty throughout the model, which is covered in more depth in Additional file 1: Appendix 8.

### Pregnancy simulation

The population structure, divided into 5-year age groups, of the simulated cohort of pregnancies is informed from demographic estimates of women and birthing people of reproductive age (10–54 years) paired with estimates of age-specific fertility rates from the GBD study at a country- and year-specific level [22]. Each simulated individual begins the simulation on day 0 of their pregnancy in our model. Possible pregnancy outcomes include live birth, stillbirth, and abortion/miscarriage, with relative frequencies informed from GBD estimates. Infant sex is determined according to estimates of live births by sex from the GBD study. Pregnancy duration is determined according to the location- and sex-specific gestational age distribution of live births in GBD for both live births and stillbirths. For pregnancies that result in abortion/miscarriage, duration is determined according to a uniform distribution between 6 and 24 weeks. Each simulated individual is also assigned a continuous hemoglobin concentration and a dichotomous exposure for prepregnancy BMI above or below 18.5, each informed from GBD risk exposure estimates. Hemoglobin and BMI exposures are correlated to one another as informed from the Woman First trial [27] (more details in Additional file 1: Appendix 2).

We advanced the simulation clock in increments of 7-day time steps and adjusted simulant ages accordingly. We used pregnancy-specific hemoglobin threshold values for anemia and simulants’ hemoglobin values to assign severity-specific anemia exposures and YLDs due to anemia accrued according to the corresponding disability weight (more details in Additional file 1: Appendix 2).

Simulated birth events occur when the simulation time clock reaches the end of a simulant’s assigned pregnancy duration. At the moment of birth, simulants experience incident nonfatal or fatal cases of pregnancy-related disorders according to the population-level age- and location-specific probability from GBD that is further modified by their hemoglobin concentration at birth. Pregnancy-related disorders in this model are defined as all conditions resulting in a loss of health tied to pregnancy, birth, or postpartum complications. A full list of the included health conditions can be found in Additional file 1: Appendix 2. Incident, nonfatal pregnancy-related disorder causes accumulate YLDs as estimated in the 2021 GBD study. In a similar manner to pregnancy-related disorders, simulants also may experience incident cases of postpartum hemorrhage at birth with a likelihood modified by their hemoglobin exposure. For simulants that experience incident cases of postpartum hemorrhage, we applied a corresponding decrease on their postpartum hemoglobin level. We assigned infant birthweight according to the joint distribution with gestational age at birth (equivalent to pregnancy duration) from GBD and further correlated with joint antenatal anemia and pre-pregnancy/first trimester BMI exposure. Details on the magnitude and data sources of these effects can be found in Additional file 1: Appendix 2.

Simulants are followed for anemia morbidity in the pregnancy simulation for an additional 6 weeks following birth before exiting the simulation. No background mortality due to causes other than pregnancy-related disorders is considered in the pregnancy simulation. Due to the limited time frame of pregnancy and relative health of the population of women of reproductive age, background mortality is negligible compared to pregnancy-related disorders.

### Childhood simulation

We initialized the simulated population of children under 5 with the birth events in the pregnancy simulation. Each live birth that occurs in the pregnancy simulation is initialized into the simulation on day 0 of their life, with values for sex, gestational age at birth, and birthweight as determined in the pregnancy simulation. Our model progresses in time steps of 4 days, and we track ages within the age groups of 0–6 days, 7–28 days, 1–5 months, 6–11 months, 12–23 months, and 2–4 years. Assigned gestational age and birthweight exposures affect mortality due to causes affected by the risk factor in the GBD 2021 study [23] for the first 28 days of life, in a manner adapted from the GBD risk effect model described in Additional file 1: Appendix 3.

Starting at 28 days of life, we assigned four-category exposure values for wasting (based on weight-for-height z-scores — WHZ), stunting (based on height-for-age z-scores — HAZ), and underweight (based on weight-for-age z-scores — WAZ). For all three metrics, we included exposure categories of severe (z-score < − 3), moderate (− 3 ≤ z-score < − 2), mild (− 2 ≤ z-score < − 1), and unaffected (z-score > − 1) with exposure prevalence informed from 2021 GBD study estimates. We additionally subdivided the moderate wasting exposure category into two substates: WHZ between − 3 and − 2.5 and WHZ between − 2.5 and − 2, in order to support the targeted MAM treatment intervention in our model. The relative exposure of each of these substates is informed from the most recently available Demographic Health Survey (DHS) data for the modeled location pooled across age groups and sexes [28], and relative morbidity and mortality risk of each substate is derived from the GBD 2021 study estimates.

We assigned each simulant a fixed stunting percentile throughout life such that a stunting exposure value may change as simulants age into the next age group with a different population-level exposure distribution, but the percentile within the age-specific population will not change. We model a dynamic transition model of child wasting that is calibrated to the GBD exposure distribution and wasting state-specific mortality rates, estimates of wasting incidence rates from longitudinal cohort studies that tracked child anthropometry in low- and low-middle-income countries (details in Additional file 1: Appendix 4), and observed recovery rates from MAM and SAM in the ComPAS trial [29] under the assumption of a steady-state equilibrium (more details in Additional file 1: Appendix 4). This calibration allows us to estimate average recovery rates from MAM and SAM states among populations without access to treatment, which are generally not available in the literature (with some exceptions [30]). Notably, wasting transition rates do not vary by moderate wasting substate exposure.

We assume no correlation between stunting and wasting exposures. Underweight exposures are assigned according to observed location-, age-, and sex-specific correlation with four-category wasting and four-category stunting obtained from the DHS and are updated dynamically as simulant age, wasting exposure, and/or stunting exposure evolve throughout the simulation (more details in Additional file 1: Appendix 4).

Infant birthweight influences population-level stunting exposures used to determine individual-level stunting exposures in accordance with evidence from the literature [31]. Infant birthweight influences wasting state at 28 days of life in accordance with observed data from DHS but does not influence wasting transition rates thereafter. More details on the impact of birthweight on child growth failure can be found in Additional file 1: Appendix 5.

We model incidence (and associated morbidity) and mortality due to diarrheal diseases, lower respiratory infections, malaria, and measles from ages 28 days to 5 years. Incidence and mortality rates due to these causes are modified by stunting, underweight, and wasting exposures (including moderate wasting substate exposures) in accordance with GBD estimates of child growth failure effects, described elsewhere [23]. Briefly, GBD methods account for overlap between these indices of child growth failure to ensure impacts on morbidity and mortality are not overestimated. We additionally model morbidity and mortality due to protein energy malnutrition, which is entirely informed by wasting exposure among simulants aged 28 days to 5 years. Finally, we model background mortality due to all causes other than those directly modeled for all ages in our child simulation.

### Intervention models

Our model considers three antenatal supplementation intervention products, including IFA, MMN, and BEP supplementation, in addition to three child nutrition interventions, including treatment for SAM, treatment for MAM, and wasting prevention with SQ-LNS.

We assume that antenatal supplementation products are distributed at routine antenatal care visits and taken for a duration of 6 months. We assume that BEP is provided in addition to MMN for pregnancies with a pre-pregnancy/first trimester BMI of less than 18.5, and otherwise antenatal supplementation products are mutually exclusive at the individual level. We additionally assume that BEP + MMN are only provided to low BMI pregnancies if MMN is also provided to adequate BMI pregnancies.

Antenatal supplementation products affect the probability of stillbirth, gestational age at birth, and birthweight, with effect sizes shown in Table 1. Intervention mean differences in antenatal hemoglobin are applied at the individual level at 8 weeks of gestation. We assume that reduction in stillbirth outcomes associated with interventions results in increases in live birth outcomes, with no changes in other birth outcomes such as abortion, miscarriage, or ectopic pregnancy. For intervention effects on preterm birth, we calculated country- and year-specific population mean differences that resulted in the relative risks reported in Table 1 and applied the mean differences to our continuous measure of gestational age at birth at the individual level in our simulation. Notably, for the effect of MMN on preterm birth, we estimated a population mean difference conditional on gestational age at birth + / − 32 weeks, such that there was a larger effect among those born at less than 32 weeks’ gestation than those born at greater than 32 weeks’ gestation so that both the effects on preterm birth (< 37 weeks) and very preterm birth (< 32 weeks) were replicated.

**Table 1:** Antenatal intervention effects

Outcome	Iron folic acid (IFA), relative to no supplementation	Multiple micronutrients (MMN), relative to IFA	Balanced energy protein (BEP), relative to MMN
**Antenatal hemoglobin, grams per liter**	*MD* = + 7.8 (4.08, 11.52) [32]	*MD* = + 0	*MD* = + 0
**Stillbirth**	*RR* = 1	*RR* = 0.91 (0.71, 0.93) [32]	*RR* = 0.39 (0.19, 0.80) [33]
**Birthweight, grams**	*MD* = + 57.73 (7.66, 107.79) [34]	*MD* = + 45.16 (32.31, 58.02) [5, 35]	*MD* = + 66.96 (13.13, 120.78) [36]^a^
**Preterm birth (< 37 weeks)**	*RR* = 0.90 (0.86, 0.95) [37]	*RR* = 0.91 (0.84, 0.99) [38]	*RR* = 1 [36]
**Very preterm birth (< 32 weeks)**	-	*RR* = 0.81 (0.71, 0.93) [35]	

*MD* mean difference, *RR* relative risk

^a^Effect specific to subpopulation of undernourished women and birthing people

In our model, the SAM treatment intervention is administered to incident cases of SAM, as defined by WHZ < − 3, between 6 and 59 months of age. In universal protocol of the MAM treatment intervention, treatment is administered to incident cases of MAM, as defined by WHZ between − 2 and − 3 and between 6 and 59 months of age. The targeted protocol of the MAM treatment intervention is administered to those eligible for the universal MAM treatment intervention who also satisfy at least one of the following criteria: (a) less than 24 months of age, (b) WHZ between − 2.5 and − 3, or (c) WAZ less than − 3. Treatment protocol and time-to-recovery for both the MAM and SAM interventions are informed from the intervention arm of the ComPAS trial, which stipulates the use of ready-to-use therapeutic food (RUTF) and regular, often weekly, monitoring and treatment until recovery [29]. We assume that a fraction of cases does not respond to treatment and recover according to the untreated recovery rates in our wasting model.

In our model, SQ-LNS supplementation begins at 6 months of age for a duration of 12 months for all infants. SQ-LNS supplementation decreases the prevalence of moderate and severe stunting and increases the prevalence of no stunting in accordance with effects obtained from study authors of an individual participant meta-analysis on the intervention [39]. We model effects of SQ-LNS supplementation on transition rates from no wasting to mild wasting, mild wasting to moderate wasting, and moderate wasting to severe wasting with no effects on wasting recovery rates. These effect sizes are calibrated to replicate the wasting prevalence ratios from the same source as stunting effect sizes, as described in Additional file 1: Appendix 6.

We assume that the initial point of care for all child nutrition interventions is monthly community management of acute malnutrition (CMAM) screenings. We assume that children are assessed for acute malnutrition and referred for treatment as appropriate at these meetings, and that SQ-LNS product counseling and distribution occurs at these meetings as well, as has been performed in trial settings [40]. Notably, this assumption dictates that for a population of 100 children 6–59 months, 5 of whom are afflicted with SAM, all 100 children would be screened in order to reach and treat the five afflicted children for SAM.

### Optimization structure

#### Scenario layout and optimization function

We ran the health model for every possible combination of our modeled interventions. We rely on the key assumption that individuals within our simulated population are independent of one another in that the health status of one individual does not impact any others. This allows us to assume that the population health status under 50% coverage of a given intervention can be equivalently represented as the average between the population health status under 100% coverage of the intervention and the population health status under 0% coverage.

In each scenario, we record counts of deaths, stillbirths, YLLs, YLDs, incident wasting cases, person-time (total time simulants spent alive), and intervention administration counts that occurred in our simulated populations. Deaths, YLLs, and YLDs are stratified by pregnancy and child populations, and person-time counts are stratified by stunting state and age group for children.

We use the *scipy.optimize* package in Python [41] to perform our allocative efficiency analysis. Inputs to our optimization function include the recorded health outcomes for each modeled scenario and the calculated cost of each scenario (obtained by multiplying the recorded intervention administration counts for each scenario by the intervention unit costs). The optimization objective is to find the fractional combination of scenarios that maximizes/minimizes the specified health quantity (e.g., minimize DALYs or maximize person-time). With this information, we calculate overall cost, health impact, and intervention coverage as outputs of our optimization function for a single budget size. Further information on the optimization constraints can be found in Additional file 1: Appendix 7.

### Illustrative example parameters

As an illustrative example, we ran the model for Ethiopia in 2021. We utilized a simulated population size of 1,600,000 pregnancies and informed the simulated child population from the resulting birth outcomes from a random sample of 400,000 of these pregnancies. These population sizes were selected as the minimum size that achieved stability in the difference between health outcomes across modeled scenarios among the antenatal and child populations, respectively. We scaled results from the pregnancy and child baseline health models by a factor of 3.5 and 14, respectively, to reflect the total estimated number of births in Ethiopia in 2021. We ran our simulation model for 20 Monte Carlo uncertainty draws (see Additional file 1: Appendix 8).

Values for baseline intervention coverage, maximum/saturation intervention coverage, and intervention costs used for this model run are displayed in Table 2. Intervention costs used in our model represent product costs with a common non-product cost multiplier. The product costs were obtained from the UNICEF Supply Catalogue for IFA, MMN, BEP, and SQ-LNS [42] interventions and the ComPAS trial [29] for MAM and SAM treatment interventions. Costs from the UNICEF Supply Catalogue were obtained in November of 2023. We assumed there were no product costs associated with CMAM screenings. Non-product costs were included through a constant 37% increase, as was used in the LiST model which represents program costs (15% increase), logistics and waste (5%), and inefficiencies (17%) [43]. We do not include service costs in this model.

**Table 2:** Intervention coverage and cost assumptions

Intervention	Baseline coverage	Saturation coverage	Product cost (2023 USD)	Final cost (2023 USD)
IFA	60.2% (95% *CI*: 48.7, 72.6) (personal communication of work by Nat Henry at CIFF)	75.7% (ANC1; GBD 2021)	US $2.27 per supplemented pregnancy	US $3.11 per supplemented pregnancy
MMN	0%, assumption	75.7% (ANC1; GBD 2021)	US $3.47 per supplemented pregnancy	US $4.75 per supplemented pregnancy
BEP	0%, assumption	75.7% (ANC1; GBD 2021)	US $40.28 per supplemented pregnancy	US $55.18 per supplemented pregnancy
SAM treatment	48.8% (95% *CI*: 37.4, 60.4) [44]	70%, assumption	US $41.84 per treated child	US $57.32 per treated child
MAM treatment	15% (95% *CI*: 10, 20), assumption. Baseline coverage assumed to be universal implementation of MAM treatment intervention	70%, assumption (applies to both universal and targeted implementations of MAM treatment intervention)	US $29.70 per treated child (applies to both universal and targeted implementations of MAM treatment intervention)	US $40.69 per treated child (applies to both universal and targeted implementations of MAM treatment intervention)
SQ-LNS	0%, assumption	70%, assumption	US $35.75 for 12-month supplementation	US $48.98 for 12-month supplementation
CMAM screening	48.8% (95% *CI*: 37.4, 60.4), assumed to be maximum of SAM treatment, MAM treatment, and SQ-LNS baseline coverage	70%, assumption	US $0 per child screened	US $0 per child screened

*IFA* iron and folic acid, *MMN* multiple micronutrients, *BEP* balanced energy protein, *SAM* severe acute malnutrition, *MAM* moderate acute malnutrition, *SQ-LNS* small quantity lipid-based nutrient supplementation, *CMAM* community management of acute malnutrition, *USD* United States dollars

We performed our allocative efficiency analysis at the estimated baseline budget size in addition to 25 evenly spaced increments between 0 and the spending required to reach saturation coverage of all interventions. We performed allocative efficiency analysis at the draw level as well as for the average result across all draws from the underlying health model.

To assess the sensitivity of these results to the assumptions in our costing model, we conducted a change-point analysis for the successive ordering of products, where we determined the change in price to each single product that would result in a change to the optimal ordering of products introduced in response to successively higher budgets.

## Results

### High-level results

**Fig. 2:**
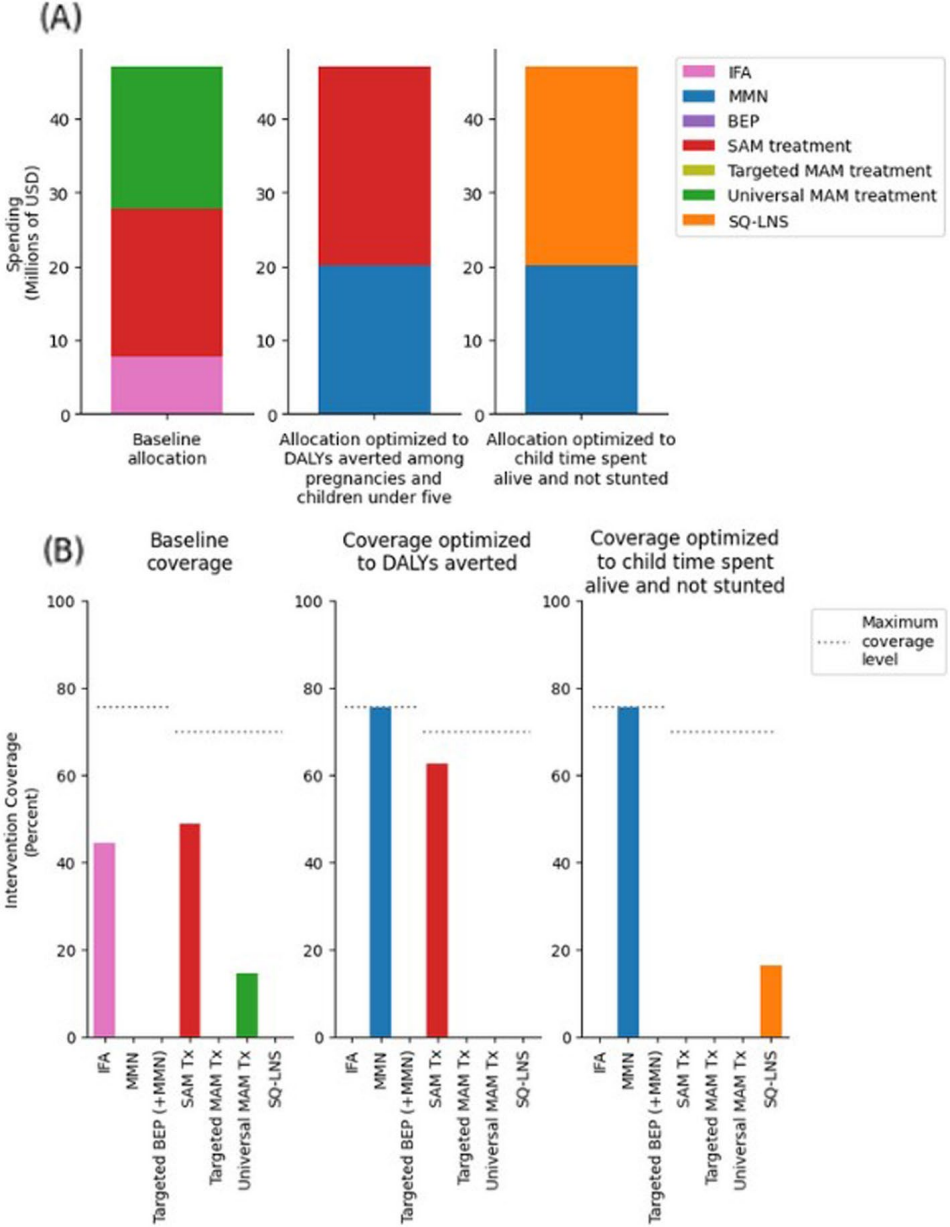
**A** Spending allocation by intervention at baseline and under optimized reallocations of the baseline budget in Ethiopia. **B** Intervention coverage at baseline and under optimized reallocations of the baseline budget in Ethiopia. IFA, iron and folic acid; MMN, multiple micronutrients; BEP, balanced energy protein; SAM, severe acute malnutrition; MAM, moderate acute malnutrition; SQ-LNS, small-quantity lipid-based nutrient supplementation; Tx, treatment; USD, United States dollars; DALY, disability-adjusted life year

From our assumed intervention coverage values and intervention costs, we estimated total annual spending on our modeled interventions to be US $47.1 (95% uncertainty interval (UI): 38.8, 58.0) million in Ethiopia, with 7.7 (95% *UI*: 6.5, 9.2) million allocated to IFA, 20.1 (95% *UI*: 15.4, 27.9) million allocated to SAM treatment, and 19.3 (95% *UI*: 12.1, 27.1) million allocated to universal MAM treatment (Fig. 2). Our model estimated 18.0 (95% *UI*: 15.1, 21.7) million annual DALYs among pregnancies and children under 5 at baseline due to the modeled childhood and pregnancy-related health conditions. This was estimated to be 986,000 (95% *UI*: 535,000, 1,588,000) DALYs fewer than the 19.0 (95% *UI*: 15.8, 22.9) million annual DALYs under the counterfactual scenario of zero spending on our modeled interventions.

### Baseline results

We found that reallocation of the baseline budget to minimize DALYs resulted in funding MMN to its maximum coverage level of 75.6%, followed by investment of the remaining budget into treatment for SAM (which attained 62.7% population coverage). When the baseline budget was reallocated to maximize child time spent not stunted, the budget was likewise first spent maximizing MMN, but then the remaining funds were allocated to SQ-LNS supplementation alone (which attained 16.4% population coverage). We maximize child time spent not stunted rather than minimizing child time stunted because minimizing child time stunted can lead to optimizing for fewer children alive. These results fit with our modeling parameters since SQ-LNS impacts stunting and is a preventive treatment, while treatment for SAM directly impacts wasting, which is more deadly for young children. Figure 2A displays the baseline and reallocated intervention-specific funding, and Fig. 2B displays the baseline and reallocated intervention-specific coverage levels.

**Fig. 3:**
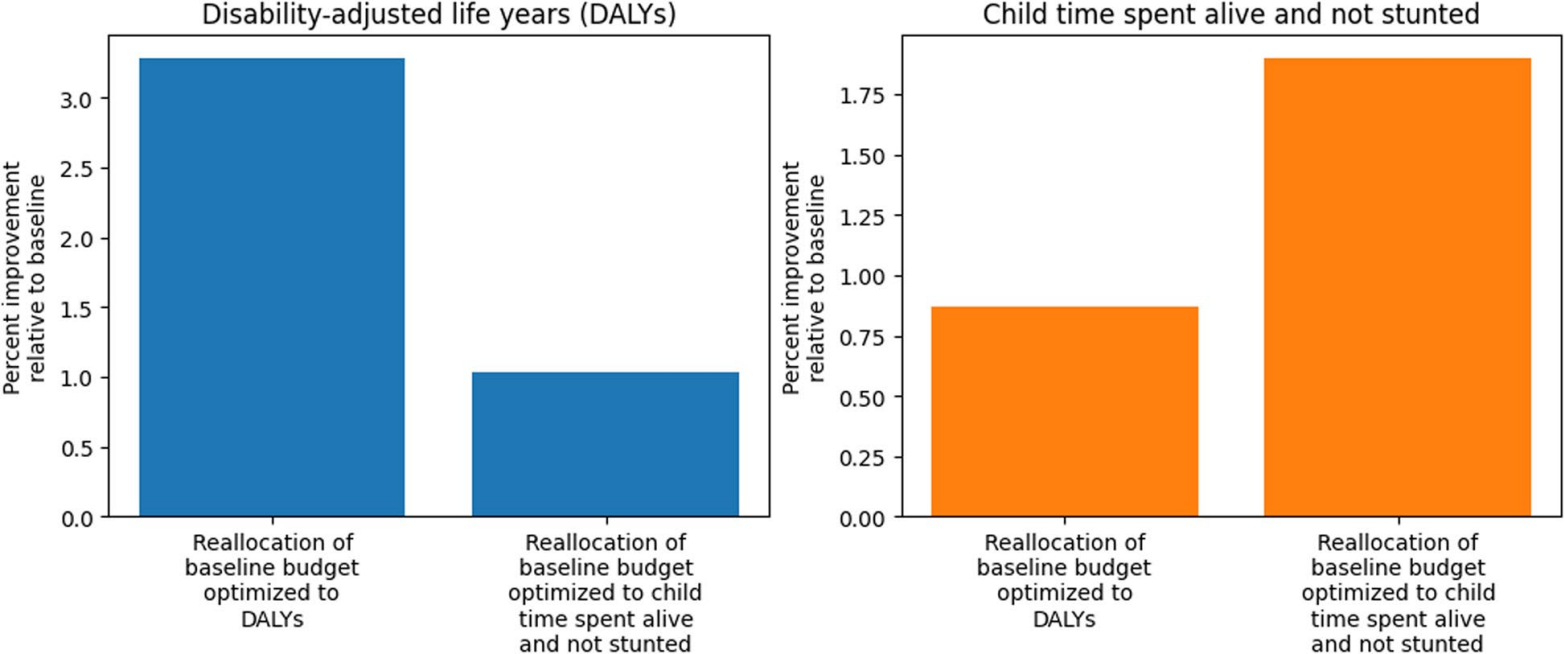
Annual disability-adjusted life years (DALYs) among pregnancies and children under 5 by scenario in Ethiopia

Relative to the baseline allocation, the reallocation optimized to minimize DALYs resulted in 592,000 fewer annual DALYs (8.3% less), and the reallocation optimized to maximize child time spent not stunted resulted in 187,000 fewer annual DALYs (6.2% less) among pregnancies and children under 5. This amounted to a 60% and 19% increase in DALYs averted relative to zero spending, compared to baseline allocation, for the reallocation optimized to DALYs, and the reallocation optimized to child time spent not stunted, respectively. Figure 3 displays annual DALYs under each of these scenarios.

### Expanding budget results

**Fig. 4:**
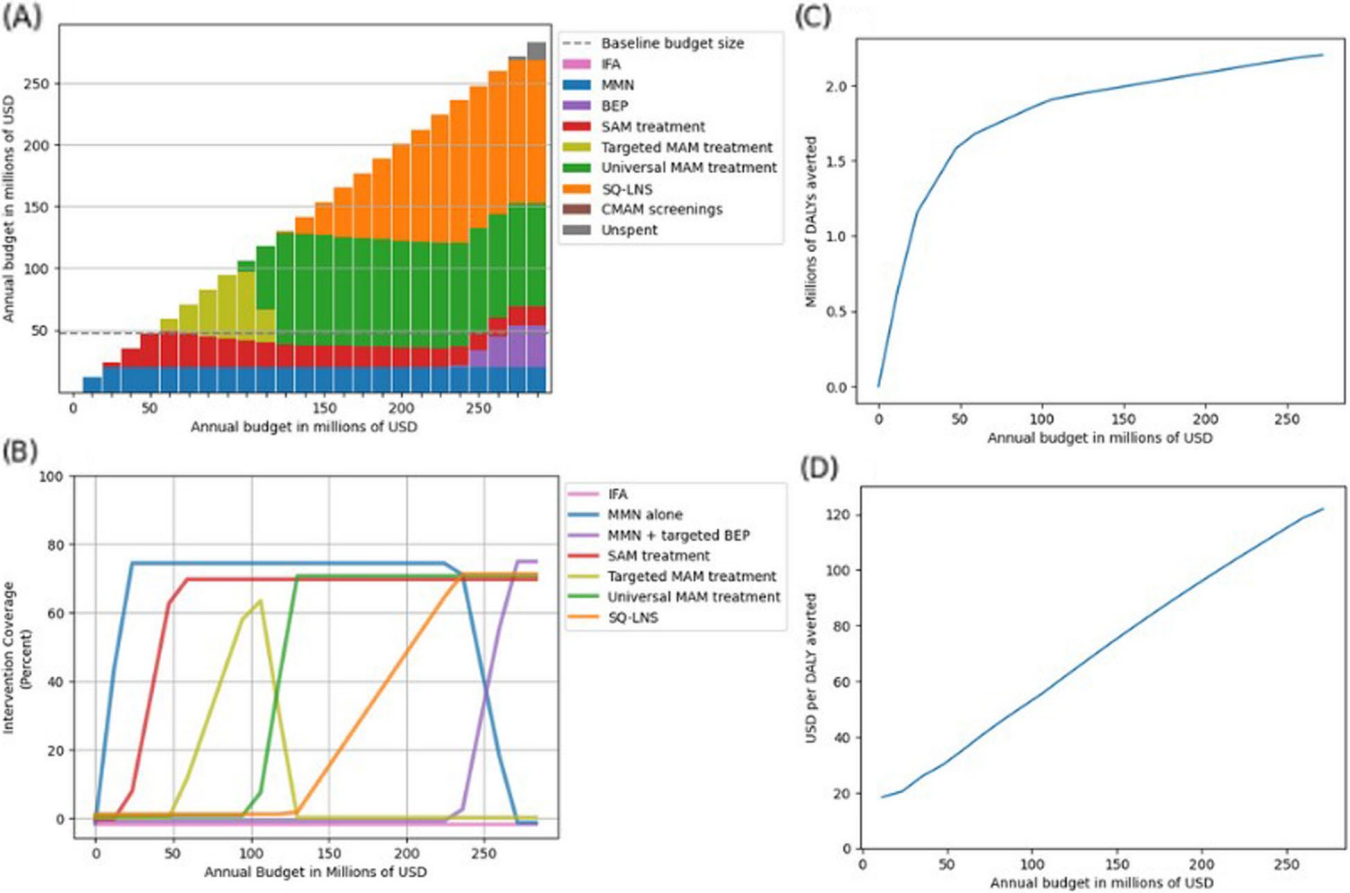
**A** Annual intervention spending allocation optimized to DALYs among pregnancies and children under 5 by increasing budget size in Ethiopia. **B** Intervention coverage optimized to DALYs among pregnancies and children under 5 by increasing budget size in Ethiopia. **C** Disability-adjusted life years (DALYs) averted among pregnancies and children under 5 relative to zero spending by increasing budget size with intervention spending allocation optimized to DALYs averted in Ethiopia. **D** Incremental cost-effectiveness ratio (ICER) relative to zero spending by increasing budget size with intervention spending allocation optimized to DALYs averted in Ethiopia. IFA, iron and folic acid; MMN, multiple micronutrients; BEP, balanced energy protein; SAM, severe acute malnutrition; MAM, moderate acute malnutrition; SQ-LNS, small-quantity lipid-based nutrient supplementation; USD, United States dollars; DALY, disability-adjusted life year

When we performed this allocative efficiency analysis for successively larger budget sizes and optimized to minimize DALYs, our model found that MMN supplementation alone should be prioritized until MMN reaches its maximum coverage level, followed by the SAM treatment intervention as budget size allows. Starting at annual budget sizes over US $50 million, our model recommends investment in the targeted MAM treatment followed by universal MAM treatment, SQ-LNS, and finally targeted BEP supplementation at the largest budget sizes. The annual budget required to achieve maximum impact (reach saturation coverage of most impactful interventions) was US $268 (95% *UI*: 226, 283) million (approximately 5.7 times more than we estimate is currently spent). Figure 4A displays the baseline and reallocated intervention-specific expenditures, and Fig. 4B displays the baseline and reallocated intervention-specific coverage levels across increasing budget sizes.

Panel C in Fig. 4 displays the annual DALYs averted relative to zero spending on any of the modeled interventions at each successive budget size, with a total of 2.2 (95% *UI*: 1.4, 3.0) million annual DALYs averted at the maximum impact level. Panel D of Fig. 4 displays the incremental cost-effectiveness ratio (ICER) in USD per DALY averted relative to zero spending on any of our modeled interventions across increasing budget sizes; note that all ICERs represented in this figure are positive with positive health gains (DALYs averted) and positive incremental costs (the northeast quadrant in as described by [45]). Notably cost-effectiveness decreases (ICER increases) with increasing budget as the most cost-effective interventions are prioritized at lower budget sizes, with all the ICER at all budget sizes at or below 121.84 (95% *UI*: 89.18, 202.70) dollars per DALY averted. Notably, without simultaneous coverage of MAM treatment or SQ-LNS interventions, US $27 (95% *UI*: 22, 37) million was required to maintain saturation coverage (70%) of the SAM treatment intervention. However, the full scale-up of MAM treatment and SQ-LNS interventions to saturation coverage (70%) reduced the operating cost of maintaining saturation coverage of the SAM treatment intervention by approximately half to US $15 (95% *UI*: 11, 23) million annually.

### Confidence and sensitivity of results

Our simulation was run with 20 draws. To understand the uncertainty in the efficacy results from the simulation, we analyzed the results at the draw level. A total of 100% of draws resulted in intervention priority ordering starting with MMN, followed by SAM treatment and targeted MAM treatment. However, the relative priority ordering of the universal MAM treatment, SQ-LNS, and BEP interventions had significant draw-level variation. Specifically, BEP was prioritized behind universal MAM treatment and SQ-LNS in only 65% of draws, and universal MAM treatment was prioritized ahead of SQ-LNS in only 70% of draws (see Additional file 1: Appendix 8 for more information on draws).

We summarize the results of our change-point analysis for all products in Table 3. We found, for example, to have SAM treatment be prioritized over MMN, and it would either need to cost US $19 or MMN would need to cost more than US $15, an over 100% change for both products. However, to have SQ-LNS change to be ahead of universal MAM, it would need to be US $44, only a 10% change in cost. Similarly, to have SQ-LNS be prioritized behind BEP, it would need to cost US $54, also about a 10% change. The change in cost of BEP is even smaller, less than US $1 to be prioritized over SQ-LNS.

**Table 3:** Costing change-point analysis of products

Product and standard cost in 2023 USD	Cost to be prioritized over prior product in 2023 USD	Cost to be prioritized behind next product in 2023 USD
IFA, 3.11 per supplemented pregnancy	US $2 — *to be prioritized over MMN at the lowest budget sizes*	N/A
MMN, 4.75 per supplemented pregnancy	N/A — *no prior product*	US $15
SAM treatment, 57.32 per treated child	US $19	US $178
Targeted MAM treatment, 40.69 per treated child	US $15	N/A — *will never be less cost effective than universal MAM due to model constraints*
Universal MAM treatment, 40.69 per treated child	N/A — *will never be more cost effective than targeted MAM due to model constraints*	US $45
SQ-LNS, 48.98 for 12-month supplementation	US $44	US $54
Targeted BEP, 55.18 per supplemented pregnancy	US $55	N/A — *no later product*

*MMN* multiple micronutrients, *IFA *iron and folic acid, *SAM *severe acute malnutrition, *MAM *moderate acute malnutrition, *SQ-LNS *small quantity lipid-based nutrient supplementation, *BEP *balanced energy protein, *USD *United States dollars

## Discussion

Our simulation provides a new model to estimate optimal allocation of spending on antenatal and child nutrition interventions utilizing a detailed health model informed from GBD study estimates that accounts for the interaction between preventive and therapeutic approaches. Our results show that an optimized reallocation of current spending on these products can substantially improve pregnancy-related and child health without additional funding and provides direction and information on the confidence of results about how to best allocate expanded budgets to maximize impact. We find very low sensitivity to the initial optimized product results, which indicates greater confidence that MMN and the treatment of SAM will be cost-effective. However, the ordering of later products is highly sensitive to changes in costs, indicating a low confidence in the results and more similar benefit between products. This is true for both efficacy and cost-sensitivity analyses.

Our model provides additional benefit relative to existing options such as the MMS cost–benefit tool [9], the CMAM costing tool [11], the FACET4SNF model [10], WBCi [12], and MINIMOD [13] in that it integrates both antenatal and child micro- and macronutrition interventions and performs allocative efficiency analysis of such interventions. Our underlying health model utilizes a similar framework as the LiST model that is utilized by Optima Nutrition, which, to the best of our knowledge, is the only other allocative efficiency model that evaluates similar interventions. However, our model differs from LiST in several ways. Firstly, LiST is a compartmental rather than microsimulation model and utilizes a variety of data sources described elsewhere [15] to inform baseline population and mortality dynamics, whereas our model primarily utilizes GBD study data. While our models generally utilize similar data sources for intervention effect estimations, they differ with respect to the derivation of MAM and SAM treatment intervention impacts on recovery rates, with our model ultimately utilizing a slightly lower effect of MAM treatment on MAM recovery and slightly higher effect of SAM treatment on SAM recovery [46]. Further, unlike our model, LiST applies intervention effects to subsets of the population only; for instance, the effect of SQ-LNS is applied to the food-insecure portion of the population only [47]. With regard to the impact of the BEP supplementation intervention, our model benefits from a correlation of the population eligible for BEP (low BMI pregnancies) with low birthweight outcomes, which is not the case in the LiST and Optima Nutrition models [46]. These differences are a result of our use of microsimulation, rather than compartmental models (as is used for LiST), in this research. Unlike our model that accounts for correlation between wasting, stunting, and underweight exposures in the attribution of their effects on morbidity and mortality, LiST does not report to account for this in the estimation of risk associated with wasting and stunting in their model. Notably, intervention protocols and definitions may slightly differ between our models. LiST also supports several interventions not included in our model [46].

Our model benefits from high-quality and detailed age-, sex-, location-, and year-specific estimates from the 2021 GBD study and framework. A strength of this data source is how it accounts for correlation between wasting, stunting, and underweight exposures in its estimation of their impacts in morbidity and mortality; this avoids overestimation of the aggregate effects. Another strength is the continuous joint distribution of birthweight and gestational age at birth. Our modeling approach allows us to represent nonlinear interactions between intervention combinations in that the presence of prevention interventions may remove the need for treatment interventions at the individual level and the optimization can allow us to understand the sensitivity of results to changes in costing. Finally, our model supports MAM treatment intervention as an independent program from SAM treatment that allows for testing of optimal coverage levels of these programs and additionally supports a targeted implementation of the MAM treatment intervention in a manner intended to reflect the recent WHO guidelines on the matter. Our model additionally supports BEP supplementation targeting to undernourished pregnancies rather than undernourished populations, which is expected to reach a greater proportion of those who may benefit from the intervention.

Our model is limited by the intensive computational resources required to run the microsimulation for our underlying health model, which we have performed on a high-performance computing cluster. Additionally, our model is limited in that it relies on assumptions and/or uncertain estimates of baseline intervention coverage and therefore baseline spending allocation. For instance, we assume that all antenatal care programs and CMAM programs through which the interventions would be delivered are fully functional up to the saturation caps.

We acknowledge that the intervention costs used in this analysis are not necessarily reflective of the true costs of implementing intervention programs at scale and are designed to provide a sense of optimization but are not designed to be used in practice as real cost estimates. One of the more notable limitations is that we do not attempt to include service costs, which LiST and other models do [48]. Our cost estimates are further limited by our assumption that the baseline budget can be reallocated effectively without additional loss of funding to inefficiencies, and by our assumption that the relationship between costing and coverage is linear. We include the same proportional increase in costing based on product costs, while in practice some interventions might require a higher or lower percent of funding for logistics and services — for example, IFA and MMN are similar products with similar distribution networks and so therefore might have more similar programmatic and logistics costs than are reflected here. Lastly, the costs included are not location specific. We hope to expand our costing strategy in future publications by utilizing programmatic, clinical trial, and GBD healthcare utilization data in order to add service costs and make estimates location specific as we plan to have multiple locations in future model versions.

Our baseline health model is limited in that it does not consider seasonality in wasting burden and relies on classification of acute malnutrition using WHZ alone rather than WHZ, mid-upper arm circumference (MUAC), and the presence of edema combined. Furthermore, our targeted MAM treatment intervention is inspired by the recent WHO guidelines but does not consider all recommended criteria for determining which MAM cases should receive treatment. Likewise, our model of BEP supplementation targeting is not exactly aligned with recommendations in the WHO guidelines. Rather than targeting at a population level, as is recommended by WHO, we utilize individual-level targeting, made possible through microsimulation. We do not model SQ-LNS intervention effects on anemia or vitamin A deficiency [49] in our model, nor do we consider a targeted implementation of the intervention such as that suggested in the WHO guideline. Finally, while our model may not represent all causal pathways by which our modeled interventions affect morbidity and mortality, as the absence of evidence between a given risk/outcome pair does not imply the absence of a causal association. Notably, we also do not consider feedback between wasting and stunting exposures (wasting leading to future stunting or vice versa) nor do we consider any causal impacts of infectious disease episodes leading to future wasting and/or stunting exposures, despite some evidence for such associations [50, 51]. The inclusion of these associations might lead to a concentration of wasting or stunting cases in certain individuals, allowing for more effective targeting. However, we do not expect the overall population estimates to be impacted considerably.

Our next steps for this model include the development of an interactive, online tool similar to the other models referenced here. This will allow user customization of the optimization and a more in-depth review of our results. Additionally, we will integrate a targeted SQ-LNS intervention implementation and perform sensitivity analyses around intervention cost assumptions, optimization measure, and location. We will also explore the robustness of intervention priority and evaluate the “next best” options. We also plan to extend this work for use in capacity planning for CMAM programs as they are adapted and/or expanded to align with updated guidelines on acute malnutrition prevention and treatment. Future extensions of this model may also include adaptation to support nonlinear intervention cost and coverage functions and/or to support subnational-level allocative efficiency analysis within a given country.

Our model enables estimation of optimal spending allocations across several antenatal and child nutrition interventions for a specified budget size using high-quality GBD study estimates and may be compared to results from the existing LiST and/or Optima Nutrition models as an exploration of structural uncertainty in the pregnancy-related and child nutrition intervention optimization space. We hope our model can aid policy makers in decisions of how to best allocate future spending on pregnancy-related and child health nutrition interventions, especially as they may be planning for integration of the recent WHO guidelines on the treatment and prevention of childhood acute malnutrition.

## Conclusions

Our simulation offers a novel approach to optimizing spending on antenatal and child health nutrition interventions, leveraging the GBD study to create a detailed health model that can more accurately account for the interaction between preventative and therapeutic approaches. In this illustrative example, by reallocating funding to MMN and SAM treatment, 592,000 DALYs could be averted annually in Ethiopia without further financial investment. The addition of this new simulation allows for greater confidence in modeling results overall and new information for key decision-makers to consider.

## Supplementary Information

Additional file 1. Extended information on methodology for the simulation and optimization. Appendix 1. Gender-inclusive language. Appendix 2. Pregnancy-related hemoglobin effects and their sources. Appendix 4. Child growth failure models. Appendix 5. Impact of birthweight on child growth failure. Appendix 6. Small-quantity lipid-based nutrient supplementation intervention effects. Appendix 7. Scenario layout, optimization function and constraints. Appendix 8. Monte Carlo draws and propagating uncertainty.

## Data Availability

The datasets generated for use during the current study are publicly available. The link for the pregnancy simulation data is: https://doi.org/10.5281/zenodo.11661268 [20] and the link for the child simulation data is: https://doi.org/10.5281/zenodo.11661095 [21].

## Abbreviations

AM: Acute malnutrition
BEP: Balanced energy protein
CMAM: Community management of acute malnutrition
DALY: Disability-adjusted life year
DHS: Demographic health survey
DW: Disability weight
GBD: Global burden of disease study
HAZ: Height-for-age z-score
ICER: Incremental cost-effectiveness ratio
IFA: Iron folic acid
MAM: Moderate acute malnutrition
MMN: Multiple micronutrient
MQ-LNS: Medium-quantity lipid-based nutrient supplement
MUAC: Mid-upper arm circumference
SAM: Severe acute malnutrition
SDG: Sustainable development goal
SQ-LNS: Small-quantity lipid-based nutrient supplement
TMREL: Theoretical minimum risk exposure level
TMRLE: Theoretical minimum risk life expectancy
WAZ: Weight-for-age z-score
WHO: World Health Organization
WHZ: Weight-for-height z-score
YLD: Years lived with disability
YLL: Years of life lost

## References

[1] The State of Food Security and Nutrition in the World 2022. FAO; 2022. Available from: http://www.fao.org/documents/card/en/c/cc0639en. Cited 2022 Jul 29.

[2] Hong Nguyen P, Singh N, Scott S, Neupane S, Jangid M, Walia M, et al. Unequal coverage of nutrition and health interventions for women and children in seven countries. Bull World Health Organ. 2022;100(1):20–9. 35017754 10.2471/BLT.21.286650 PMC8722629

[3] World Health Organization. WHO antenatal care recommendations for a positive pregnancy experience Nutrition interventions update: multiple micronutrient supplements during pregnancy. 2020. Available from: https://iris.who.int/bitstream/handle/10665/333561/9789240007789-eng.pdf?sequence=1. 32783435

[4] World Health Organization. WHO recommendations on antenatal care for a positive pregnancy experience. 2016. Available from: https://iris.who.int/bitstream/handle/10665/250796/9789241549912-eng.pdf?sequence=1. 28079998

[5] Young N, Bowman A, Swedin K, Collins J, Blair-Stahn ND, Lindstedt PA, et al. Cost-effectiveness of antenatal multiple micronutrients and balanced energy protein supplementation compared to iron and folic acid supplementation in India, Pakistan, Mali, and Tanzania: a dynamic microsimulation study. Myers JE, editor. PLOS Med. 2022;19(2):e1003902. 35192606 10.1371/journal.pmed.1003902 PMC8863292

[6] World Health Organization. WHO guideline on the prevention and management of wasting and nutritional oedema (acute malnutrition) in infants and children under 5 years. 2023. Available from: https://app.magicapp.org/#/guideline/noPQkE. 38498638

[7] Heidkamp RA, Piwoz E, Gillespie S, Keats EC, D’Alimonte MR, Menon P, et al. Mobilising evidence, data, and resources to achieve global maternal and child undernutrition targets and the Sustainable Development Goals: an agenda for action. Lancet. 2021;397(10282):1400–18. 33691095 10.1016/S0140-6736(21)00568-7

[8] Ruel MT, Menon P, Habicht JP, Loechl C, Bergeron G, Pelto G, et al. Age-based preventive targeting of food assistance and behaviour change and communication for reduction of childhood undernutrition in Haiti: a cluster randomised trial. Lancet. 2008;371(9612):588–95. 18280329 10.1016/S0140-6736(08)60271-8

[9] Verney AMJ, Busch-Hallen JF, Walters DD, Rowe SN, Kurzawa ZA, Arabi M. Multiple micronutrient supplementation cost–benefit tool for informing maternal nutrition policy and investment decisions. Matern Child Nutr. 2023;19(4):e13523. 37378454 10.1111/mcn.13523 PMC10483938

[10] The food assistance cost-effectiveness tool for specialized nutritious foods. Available from: https://foodaidquality.nutrition.tufts.edu/focus/cost-effectiveness-tools.

[11] CMAM Costing Tool. Available from: https://www.fantaproject.org/tools/cmam-costing-tool.

[12] Holla-Bhar R, Iellamo A, Gupta A, Smith JP, Dadhich JP. Investing in breastfeeding – the world breastfeeding costing initiative. Int Breastfeed J. 2015;10(1):8. 25873985 10.1186/s13006-015-0032-y PMC4396713

[13] Brown KH, Engle-Stone R, Kagin J, Rettig E, Vosti SA. Use of optimization modeling for selecting national micronutrient intervention strategies: an example based on potential programs for control of vitamin A deficiency in Cameroon. Food Nutr Bull. 2015;36(3_suppl):S141-8. 26283708 10.1177/0379572115599325

[14] Pearson R, Killedar M, Petravic J, Kakietek JJ, Scott N, Grantham KL, et al. Optima nutrition: an allocative efficiency tool to reduce childhood stunting by better targeting of nutrition-related interventions. BMC Public Health. 2018;18(1):384. 29558915 10.1186/s12889-018-5294-z PMC5861618

[15] Walker N, Tam Y, Friberg IK. Overview of the Lives Saved Tool (LiST). BMC Public Health. 2013;13(Suppl 3):S1. 24564438 10.1186/1471-2458-13-S3-S1 PMC3847271

[16] Baldissera PM. Structural uncertainty through the lens of model building. Synthese. 2021;198(11):10377–93.

[17] Vivarium. Available from: https://vivarium.readthedocs.io/en/latest/.

[18] Haddock B, Pletcher A, Blair-Stahn N, Keyes O, Kappel M, Bachmeier S, et al. Simulated data for census-scale entity resolution research without privacy restrictions: a large-scale dataset generated by individual-based modeling. Gates Open Res. 2024;3(8):36. 10.12688/gatesopenres.15418.2 PMC11518969 39474508

[19] Kannan A, Tsoi D, Xie Y, Horst C, Collins J, Flaxman A. Cost-effectiveness of vitamin A supplementation among children in three sub-Saharan African countries: an individual-based simulation model using estimates from Global Burden of Disease 2019. Horton S, editor. PLOS ONE. 2022;17(4):e0266495. 35390077 10.1371/journal.pone.0266495 PMC8989187

[20] Mudambi R, Jafari H, Albright J, Nast P, Lutze S, Bowman A, et al. ihmeuw/vivarium_gates_nutrition_optimization: archival release. Zenodo; 2024. Available from: https://zenodo.org/records/11661268. Cited 2024 Dec 6.

[21] Mudambi R, Jafari H, Albright J, Nast P, Lutze S, Bowman A, et al. ihmeuw/vivarium_gates_nutrition_optimization_child: archival release. Zenodo; 2024. Available from: https://zenodo.org/records/11661095. Cited 2024 Dec 6.

[22] Bhattacharjee NV, Schumacher AE, Aali A, Abate YH, Abbasgholizadeh R, Abbasian M, et al. Global fertility in 204 countries and territories, 1950–2021, with forecasts to 2100: a comprehensive demographic analysis for the Global Burden of Disease Study 2021. The Lancet. 2024;403(10440):2057–99. 10.1016/S0140-6736(24)00550-6 PMC11122687 38521087

[23] Brauer M, Roth GA, Aravkin AY, Zheng P, Abate KH, Abate YH, et al. Global burden and strength of evidence for 88 risk factors in 204 countries and 811 subnational locations, 1990–2021: a systematic analysis for the Global Burden of Disease Study 2021. The Lancet. 2024;403(10440):2162–203. 10.1016/S0140-6736(24)00933-4 PMC11120204 38762324

[24] Ferrari AJ, Santomauro DF, Aali A, Abate YH, Abbafati C, Abbastabar H, et al. Global incidence, prevalence, years lived with disability (YLDs), disability-adjusted life-years (DALYs), and healthy life expectancy (HALE) for 371 diseases and injuries in 204 countries and territories and 811 subnational locations, 1990–2021: a systematic analysis for the Global Burden of Disease Study 2021. The Lancet. 2024;403(10440):2133–61. 10.1016/S0140-6736(24)00757-8 PMC11122111 38642570

[25] Naghavi M, Ong KL, Aali A, Ababneh HS, Abate YH, Abbafati C, et al. Global burden of 288 causes of death and life expectancy decomposition in 204 countries and territories and 811 subnational locations, 1990–2021: a systematic analysis for the Global Burden of Disease Study 2021. The Lancet. 2024;403(10440):2100–32. 10.1016/S0140-6736(24)00367-2 PMC11126520 38582094

[26] Schumacher AE, Kyu HH, Aali A, Abbafati C, Abbas J, Abbasgholizadeh R, et al. Global age sex-specific mortality, life expectancy, and population estimates in 204 countries and territories and 811 subnational locations, 1950–2021, and the impact of the COVID-19 pandemic: a comprehensive demographic analysis for the Global Burden of Disease Study 2021. The Lancet. 2024;403(10440):1989–2056. 10.1016/S0140-6736(24)00476-8 PMC11126395 38484753

[27] Hambidge KM, Westcott JE, Garcés A, Figueroa L, Goudar SS, Dhaded SM, et al. A multicountry randomized controlled trial of comprehensive maternal nutrition supplementation initiated before conception: the Women First trial. Am J Clin Nutr. 2019;109(2):457–69. 30721941 10.1093/ajcn/nqy228 PMC6367966

[28] Ethiopian Public Health Institute - EPHI, Federal Ministry of Health - FMoH, and ICF. 2021. Ethiopia Mini Demographic and Health Survey 2019. Addis Ababa, Ethiopia: EPHI/FMoH/ICF. https://www.dhsprogram.com/pubs/pdf/FR363/FR363.pdf.

[29] Bailey J, Opondo C, Lelijveld N, Marron B, Onyo P, Musyoki EN, et al. A simplified, combined protocol versus standard treatment for acute malnutrition in children 6–59 months (ComPAS trial): a cluster-randomized controlled non-inferiority trial in Kenya and South Sudan. Tumwine JK, editor. PLOS Med. 2020;17(7):e1003192. 32645109 10.1371/journal.pmed.1003192 PMC7347103

[30] James P, Sadler K, Wondafrash M, Argaw A, Luo H, Geleta B, et al. Children with moderate acute malnutrition with no access to supplementary feeding programmes experience high rates of deterioration and no improvement: results from a prospective cohort study in rural Ethiopia. PLoS ONE. 2016;11(4):e0153530. 27100177 10.1371/journal.pone.0153530 PMC4839581

[31] McGovern ME. How much does birth weight matter for child health in developing countries? Estimates from siblings and twins. Health Econ. 2019;28(1):3–22. 30239053 10.1002/hec.3823

[32] Oh C, Keats E, Bhutta Z. Vitamin and mineral supplementation during pregnancy on maternal, birth, child health and development outcomes in low- and middle-income countries: a systematic review and meta-analysis. Nutrients. 2020;12(2):491. 32075071 10.3390/nu12020491 PMC7071347

[33] Lassi ZS, Padhani ZA, Rabbani A, Rind F, Salam RA, Das JK, et al. Impact of dietary interventions during pregnancy on maternal, neonatal, and child outcomes in low- and middle-income countries. Nutrients. 2020;12(2):531. 32092933 10.3390/nu12020531 PMC7071393

[34] Peña-Rosas JP, De-Regil LM, Gomez Malave H, Flores-Urrutia MC, Dowswell T. Intermittent oral iron supplementation during pregnancy. Cochrane Pregnancy and Childbirth Group, editor. Cochrane Database Syst Rev. 2015;2015(10). Available from: http://doi.wiley.com/10.1002/14651858.CD009997.pub2. Cited 2024 Jan 19. 10.1002/14651858.CD009997.pub2 PMC7092533 26482110

[35] Keats EC, Haider BA, Tam E, Bhutta ZA. Multiple-micronutrient supplementation for women during pregnancy. Cochrane Pregnancy and Childbirth Group, editor. Cochrane Database Syst Rev. 2019; Available from: https://doi.wiley.com/10.1002/14651858.CD004905.pub6. Cited 2024 Jan 19. 10.1002/14651858.CD004905.pub6 PMC6418471 30873598

[36] Ota E, Hori H, Mori R, Tobe-Gai R, Farrar D. Antenatal dietary education and supplementation to increase energy and protein intake. Cochrane Pregnancy and Childbirth Group, editor. Cochrane Database Syst Rev. 2015;2015(6). Available from: http://doi.wiley.com/10.1002/14651858.CD000032.pub3. Cited 2024 Jan 19. 10.1002/14651858.CD000032.pub3 PMC12634316 26031211

[37] Li B, Zhang X, Peng X, Zhang S, Wang X, Zhu C. Folic acid and risk of preterm birth: a meta-analysis. Front Neurosci. 2019;28(13):1284. 10.3389/fnins.2019.01284 PMC6892975 31849592

[38] Gomes F, Askari S, Black RE, Christian P, Dewey KG, Mwangi MN, et al. Antenatal multiple micronutrient supplements versus iron-folic acid supplements and birth outcomes: analysis by gestational age assessment method. Matern Child Nutr. 2023;19(3):e13509. 37002655 10.1111/mcn.13509 PMC10262881

[39] Dewey KG, Stewart CP, Wessells KR, Prado EL, Arnold CD. Small-quantity lipid-based nutrient supplements for the prevention of child malnutrition and promotion of healthy development: overview of individual participant data meta-analysis and programmatic implications. Am J Clin Nutr. 2021;114:3S-14S. 34590696 10.1093/ajcn/nqab279 PMC8560310

[40] Huybregts L, Le Port A, Becquey E, Zongrone A, Barba FM, Rawat R, et al. Impact on child acute malnutrition of integrating small-quantity lipid-based nutrient supplements into community-level screening for acute malnutrition: acluster-randomized controlled trial in Mali. Persson LÅ, editor. PLOS Med. 2019;16(8):e1002892. 31454356 10.1371/journal.pmed.1002892 PMC6711497

[41] Virtanen P, Gommers R, Oliphant TE, Haberland M, Reddy T, Cournapeau D, et al. SciPy 1.0: fundamental algorithms for scientific computing in Python. Nat Methods. 2020;17(3):261–72. 32015543 10.1038/s41592-019-0686-2 PMC7056644

[42] UNICEF Supply Catalogue. Available from: https://supply.unicef.org/. Cited 2024 Feb 5.

[43] The Lives Saved Tool. Presentations. Available from: https://www.livessavedtool.org/presentations. Cited 2024 Sep 20.

[44] Isanaka S, Andersen CT, Cousens S, Myatt M, Briend A, Krasevec J, et al. Improving estimates of the burden of severe wasting: analysis of secondary prevalence and incidence data from 352 sites. BMJ Glob Health. 2021;6(3):e004342. 33653730 10.1136/bmjgh-2020-004342 PMC7929878

[45] Klok RM, Postma MJ. Four quadrants of the cost-effectiveness plane: some considerations on the south-west quadrant. Expert Rev Pharmacoecon Outcomes Res. 2004;4(6):599–601. 19807531 10.1586/14737167.4.6.599

[46] Optima | Nutrition | Documents. Available from: https://optimamodel.com/nutrition/documents.html. Cited 2024 Dec 6.

[47] Tong H, Piwoz E, Ruel MT, Brown KH, Black RE, Walker N. Maternal and child nutrition in the lives saved tool: results of a recent update. J Glob Health. 2022;30(12):08005. 10.7189/jogh.12.08005 PMC9801341 36583418

[48] Bollinger LA, Sanders R, Winfrey W, Adesina A. Lives Saved Tool (LiST) costing: a module to examine costs and prioritize interventions. BMC Public Health. 2017;17(4):782. 29143622 10.1186/s12889-017-4738-1 PMC5688490

[49] Wessells KR, Arnold CD, Stewart CP, Prado EL, Abbeddou S, Adu-Afarwuah S, et al. Characteristics that modify the effect of small-quantity lipid-based nutrient supplementation on child anemia and micronutrient status: an individual participant data meta-analysis of randomized controlled trials. Am J Clin Nutr. 2021;114:68S-94S. 34590114 10.1093/ajcn/nqab276 PMC8560313

[50] Thurstans S, Sessions N, Dolan C, Sadler K, Cichon B, Isanaka S, et al. The relationship between wasting and stunting in young children: a systematic review. Matern Child Nutr. 2022;18(1). Available from: https://onlinelibrary.wiley.com/doi/10.1111/mcn.13246. Cited 2022 Sep 15. 10.1111/mcn.13246 PMC8710094 34486229

[51] Troeger C, Colombara DV, Rao PC, Khalil IA, Brown A, Brewer TG, et al. Global disability-adjusted life-year estimates of long-term health burden and undernutrition attributable to diarrhoeal diseases in children younger than 5 years. Lancet Glob Health. 2018;6(3):e255–69. 29433665 10.1016/S2214-109X(18)30045-7 PMC5861379

