# Supplementary material for: Optimal allocation of antenatal and young child nutrition interventions: an individual-based global burden of disease calibrated microsimulation
Source: BMC Glob Public Health. 2025 Jan 15;3:6. doi: 10.1186/s44263-024-00120-y (PMC11737270; doi:10.1186/s44263-024-00120-y)
Supplement: Supplementary file 1 — Additional file 1. Extended information on methodology for the simulation and optimization. Appendix 1. Gender-inclusive language. Appendix 2. Pregnancy-related hemoglobin effects and their sources. Appendix 4. Child growth failure models. Appendix 5. Impact of birthweight on child growth failure. Appendix 6. Small-quantity lipid-based nutrient supplementation intervention effects. Appendix 7. Scenario layout, optimization function and constraints. Appendix 8. Monte Carlo draws and propagating uncertainty. [file 44263_2024_120_MOESM1_ESM.docx]

# Additional File 1 for Bowman et al., 2024

## Appendix 1: Gender-inclusive language

Philosophy around gender-inclusive language

Our team believes inclusive language is important, and we want to begin by acknowledging the potential harm that using simplistic and gendered language can create. People who are intersex, transgender, nonbinary, and/or have other gender-nonconforming identities experience harm from gendered language in a number of ways, including documented reductions in interactions with health care, not being included in research, and lost opportunities. For that reason, we want to ensure our language moves beyond the implied gender binary in words like “maternal” or “mother”.

However, we also recognize that others, particularly those fighting for women’s empowerment, might choose to use different language to advocate for other groups. The health of women and girls can often be sidelined, and centering their experiences and needs is important and vital work. This paper, in fact, found that the best way to support newborns was to support their parents during pregnancy. That is why we strive for gender-inclusive language throughout this paper, including when possible both women-centering language and language inclusive to all people and identities.

Below is a table including some of the language commonly used in this space and our gender-inclusive alternatives, which are used throughout this paper. In some cases, we changed the language found in other researchers’ papers or methods to match our preferred, gender-inclusive options. These instances are explicitly called out below.

| **Gendered language** | **Gender-inclusive language** | **Source material changed** |
| --- | --- | --- |
| Maternal and child health | Pregnancy-related and child health | Minor references in documents for alternative optimization models |
| Maternal disorders | Pregnancy-related disorders | GBD (Naghavi et al., 2024) |
| Women of reproductive age | Women and birthing people of reproductive age | Minor references in documents for alternative optimization models |
| Maternal hemorrhage | Birth-related hemorrhage | GBD (Naghavi et al., 2024) |
| Maternal sepsis and other maternal infections | Pregnancy-related sepsis and other pregnancy-related infections | GBD (Naghavi et al., 2024) |
| Maternal hypertensive disorders | Pregnancy-related hypertensive disorders | GBD (Naghavi et al., 2024) |
| Maternal obstructed labor and uterine rupture | Birth-related obstructed labor and uterine rupture | GBD (Naghavi et al., 2024) |
| Indirect maternal deaths | Indirect pregnancy-related deaths | GBD (Naghavi et al., 2024) |
| Late maternal deaths | Late pregnancy-related deaths | GBD (Naghavi et al., 2024) |
| Maternal deaths aggravated by HIV/AIDS | Pregnancy-related deaths aggravated by HIV/AIDS | GBD (Naghavi et al., 2024) |

## Appendix 2: Pregnancy-related hemoglobin effects and their sources

Hemoglobin exposure

Hemoglobin exposure was informed from location-, year-, sex-, and age-specific estimates of mean and standard deviation hemoglobin as well as assumptions regarding distribution shape from the 2021 GBD study. More details on how these estimates were derived can be found at (Brauer et al., 2024). As our simulated population consisted entirely of pregnant women and birthing people and those up to six weeks postpartum, we adjusted the GBD hemoglobin parameters specific to women and birthing people of reproductive age to represent hemoglobin parameters among the pregnant and lactating population in accordance with the “pregnancy adjustment factor” utilized in the GBD study (Brauer et al., 2024).

Hemoglobin, body mass index, and birthweight

Population-level hemoglobin as well as population-level exposure of body mass index (BMI) < 18.5 kilograms per square meter are informed from 2021 GBD study parameters. We model a correlation between these two exposures such that those with a hemoglobin concentration less than 10 grams per deciliter (g/dL) had 2.07 (95% CI: 1.79, 2.39) times the rate of BMI exposure < 18.5 than those with hemoglobin concentration of 10 g/dL or more. The magnitude of this association was informed from Woman First trial data (Hambidge et al., 2019). We additionally model a correlation between joint anemia (hemoglobin +/- 10 g/dL) and BMI (+/- 18) exposures with infant birthweight in accordance with the mean differences displayed in the table below, obtained from Woman First trial data (Hambidge et al., 2019). Notably, the total subject count in the trial was 2,668, and 2,522 subjects had non-missing values for BMI, hemoglobin, and infant birthweight. Preference for the timepoint of hemoglobin assessment in this analysis was second trimester, followed by first trimester, and finally third trimester. Preference for the timepoint of BMI exposure assessment was pre-pregnancy date closest to conception followed by first trimester assessment.

| **Pre-pregnancy or first trimester body mass index** | **Hemoglobin during pregnancy** | **N** | **Birthweight mean difference (grams) relative to BMI** ≥ **18.5 and hemoglobin** ≥ **10 g/dL and 95% confidence intervals** |
| --- | --- | --- | --- |
| < 18.8 | < 10 g/dL | 255 | -275 (-336, -213) |
| < 18.5 | ≥ 10 g/dL | 297 | -182 (-239, -125) |
| ≥ 18.5 | < 10 g/dL | 484 | -94 (-142, -46) |
| ≥ 18.5 | ≥ 10 g/dL | 1486 | - |

Hemoglobin and anemia

Anemia status during pregnancy and through six weeks postpartum was assessed according to hemoglobin concentration and corresponding thresholds shown in the table below as informed by the World Health Organization thresholds for pregnant women and birthing people aged 15 years and older. Time spent classified in a given anemia severity resulted in accumulation of years lived with disability (YLDs) due to anemia in accordance with the disability weights shown in the table below.

| **Anemia severity** | **Hemoglobin threshold (grams per liter)** | **Disability weight** |
| --- | --- | --- |
| None | 100+ | 0 |
| Mild | 100-109 | 0.004 |
| Moderate | 70-99 | 0.052 |
| Severe | <70 | 0.149 |

Pregnancy-related disorders and hemoglobin

The population-level probability of incident and fatal pregnancy-related disorders cases per pregnancy were informed from location-, year-, sex-, and age-specific estimates from the GBD 2021 study paired with estimates of pregnancy counts, informed from 2021 GBD study location-, year-, sex-, and age-specific fertility rate estimates, location- and year-specific stillbirth to live birth ratios, and location-, year-, sex-, and age-specific estimates of abortion/miscarriage cases as well as ectopic pregnancy cases. Notably, the pregnancy-related disorders cause in the GBD 2021 study included birth-related hemorrhage, pregnancy-related sepsis and other pregnancy-related infections, pregnancy-related hypertensive disorders, birth-related obstructed labor and uterine rupture, abortion and miscarriage, ectopic pregnancy, indirect pregnancy-related deaths, late pregnancy-related deaths, pregnancy-related deaths aggravated by HIV/AIDS, and other pregnancy-related disorders. We modeled pregnancy-related disorders as a single aggregate cause of morbidity and mortality in our simulation.

We then modified these population-level rates for individual simulants according to their hemoglobin concentration at the moment of birth. We assumed that each gram per deciliter (g/dL) increase in hemoglobin concentration above 12 g/dL was associated with an increased risk of incident and fatal cases of pregnancy-related disorders equal in magnitude to the risk effect utilized in the GBD 2021 study. Notably, the risk exposure in our simulation was hemoglobin, whereas the GBD 2021 study reports burden attributable specifically to iron deficiency and performs additional analytic steps to derive these estimates. Due to this discrepancy, we calculated custom population attributable fraction values to calibrate the baseline rate of pregnancy-related disorders in our simulated population such that we replicated expected risk effects due to hemoglobin and the expected population-level rate of pregnancy-related disorders.

Fatal cases of pregnancy-related disorders accumulated years of life lost (YLLs) in accordance with their age at death and the 2021 GBD study age-specific theoretical minimum risk exposure level value. Nonfatal cases of pregnancy-related disorders accumulated years lived with disability (YLDs) in accordance with location-, year-, sex-, and age-specific estimates of YLDs due to pregnancy-related disorders (excluding any YLDs caused by anemia such as YLDs due to anemia caused by birth-related hemorrhage, as those were accounted for separately through the hemoglobin, anemia, and hemorrhage components of our model) scaled to estimates of nonfatal pregnancy-related disorders cases in the same demographic groups from the GBD 2021 study.

Postpartum hemorrhage and hemoglobin

In addition to the morbidity and mortality due to pregnancy-related disorders as described in the previous section, we also modeled probability of experiencing postpartum hemorrhage at birth in our simulation. In our model, postpartum hemorrhage had no associated morbidity and mortality (as it was already included in the aggregate pregnancy-related disorders cause of morbidity and mortality) and was included specifically to model the causal pathway between antenatal hemoglobin and postpartum hemoglobin as mediated through postpartum hemorrhage. The population-level probability of postpartum hemorrhage was informed from location-, year-, sex-, and age-specific estimates of birth-related hemorrhage incidence from the GBD 2021 study paired with estimates of pregnancy counts (as described in the above section). We assigned severity as moderate or severe to cases of postpartum hemorrhage in accordance with GBD 2021 sequelae-level estimates.

We assumed that pregnancies with hemoglobin concentrations less than 70 grams per liter (g/L) at the time of birth had a 3.54 (95% CI: 1.2, 10.4) times greater risk of postpartum hemorrhage than pregnancies with hemoglobin concentrations greater than 70 g/L at the time of birth (Omotayo et al., 2021).

For simulants who experienced postpartum hemorrhage at birth, we modeled a corresponding decrease in their postpartum hemoglobin concentration. Under the assumption that plasma volume during pregnancy increases by approximately 50% in the late third trimester (Aguree & Gernand, 2019) and an assumed blood volume of 5 liters among non-pregnant individuals (Sharma & Sharma, 2023), we assumed a blood volume of 7.5 liters at the time of birth in our simulation. We additionally assumed a blood loss of 0.75 liters for moderate hemorrhage (defined as 0.5 to 1 liters of blood loss in the GBD 2021 study) and a blood loss of 1.25 liters for severe hemorrhage (defined as >1 liter of blood loss in the GBD 2021 study). Therefore, we applied 10% and 16.7% reductions in postpartum hemoglobin concentration relative to hemoglobin concentration at birth for moderate and severe hemorrhage cases, respectively. The reduction in postpartum hemoglobin associated with postpartum hemorrhage persisted for the entirety of the six-week postpartum period tracked in our simulation.

## Appendix 3: LBWSG continuous risk interpolation details

Methods for GBD 2021 study modeling strategy for risk exposure and risk effects of infant birthweight and gestational age at birth (BW/GA) are described elsewhere (Brauer et al., 2024). Briefly, a joint continuous distribution of BW/GA is discretized into categories of 500-gram BW by two-week GA bins (with the exception of 0–24 weeks, 36–37, and 37–38 weeks). Relative risk of mortality during the neonatal period was then calculated for each exposure category relative to the categories with the lowest risk of mortality (38–40 weeks/4000–4500 grams, 38–40 weeks/3500–4000 grams, 40–42 weeks/4000–4500 grams, and 40–42 weeks/3500–4000 grams). To avoid concerns regarding reverse causality (such as congenital birth defects leading to preterm birth and/or low birthweight rather than or in addition to preterm birth and/or low birthweight leading to increased risk of neonatal death due to congenital birth defects), these relative risks were then applied to a subset of causes with biological plausibility of causality, including diarrheal diseases, lower respiratory infections, upper respiratory infections, otitis media, meningitis, encephalitis, neonatal preterm birth, neonatal encephalopathy due to birth asphyxia and trauma, neonatal sepsis and other neonatal infections, hemolytic disease and other neonatal jaundice, other neonatal disorders, and sudden infant death syndrome.

We model a joint distribution of BW/GA as informed by location-, sex-, and year-specific categorical exposure estimates from the GBD 2021 study. We convert this categorical exposure into a continuous joint exposure under the assumption of a uniform distribution within each exposure category.

We additionally convert the categorical mortality risks from the GBD 2021 study to a continuous risk surface across BW and GA exposures. This allows us to estimate the difference in mortality risk associated with a change in BW and/or GA *within* a given category rather than only among those that result in a change in categorical exposures. We do this by utilizing the *scipy.interpolate* Python package (Virtanen et al., 2020). To do this, we defined a rectangular grid of BW (0 to 4500 grams) and GA (0 to 42 weeks) and placed the log-transformed categorical relative risk value from the GBD 2021 study to represent the midpoint of each rectangular BW/GA exposure category. Notably, GBD does not model exposure categories represented on the full grid of BW/GA as many, such as 0–500 grams birthweight/40–42 weeks’ gestation have zero or near-zero observed prevalence; we utilized the nearest-neighbor interpolation method to extrapolate values of log(RR) to the midpoints of each of these “missing” categories. We use this same strategy to extrapolate log(RR) values for the intersections of birthweight boundaries (0 and 4500 grams) with the midpoint gestational age value of each BW/GA exposure category and vice versa. We then utilized the bilinear interpolation method to extrapolate log(RR) values for the remaining continuous grid points between the categorical midpoint values. We then exponentiate our extrapolated log(RR) grid values and manually reset all points within the four pre-defined lowest mortality risk categories to 1. We calculate population attributable fraction (PAF) values according to our custom-generated continuous risk surface rather than rely on GBD 2021 study PAF values generated from categorical risks. We modeled a risk effect of BW/GA on mortality due to the same causes affected by BW/GA in the GBD 2021 study.

## Appendix 4: Child growth failure models

Correlation between child growth failure metrics

We informed correlation between stunting, wasting, and underweight from the most recently available Demographic and Health Surveys (DHS) results for our modeled location. We assessed correlation between continuous measures of weight for height z-scores (WHZ), height for age z-scores (HAZ), and weight for age z-scores (WAZ) at the age-specific level with age groups of 1–5 months, 6–11 months, 12–23 months, and 1–4 years. We observed moderate (0.4 to 0.59) to strong (0.6 to 0.79) positive Spearman correlation coefficients between WAZ and HAZ as well as WAZ and WHZ for all age groups assessed. The Spearman correlation coefficient between HAZ and WHZ ranged from a weak negative correlation (-0.2 to -0.39) in the 1–5-month age group to a weak positive correlation (0.2 to 0.39) in the 12–23-month age group.

Given the weak magnitude of association between stunting and wasting and the variation across age groups, we assumed there was zero correlation between WHZ/wasting and HAZ/stunting in our simulation in order to simplify our model. We did, however, model the correlation between WAZ/underweight and HAZ/stunting as well as WAZ/underweight and WHZ/wasting in accordance with the observed age-specific values from DHS through our modeling strategy of underweight risk exposure in our simulation. We generated 500 draws of observed correlation coefficients based on confidence intervals calculated from the sample size of the data used to derive the correlation coefficients.

To achieve this, we initialize a simulated population of 150,000 individuals at the location-, age-, and sex-specific level. We assign each simulant propensity values between zero and 1 for HAZ, WAZ, and WHZ scores such that the population-level correlation between HAZ, WAZ, and WHZ propensities matched the correlation values described above. We then assign four-category stunting, underweight, and wasting exposure values to each simulated individual by benchmarking the HAZ, WAZ, and WHZ propensity values to the four-category location-, sex-, age-, and year-specific population stunting, underweight, and wasting exposure distributions from the GBD 2021 study (note that we did not consider moderate wasting substates in this portion of the analysis). We then calculate four-category underweight exposure distributions conditional on joint four-category wasting and four-category wasting stunting exposure values. For instance, the exposure prevalence of severe underweight may be 90% among those who are both severely wasted and severely stunted, whereas it may be 5% among those who are mildly wasted and mildly stunted. The underweight exposure distributions conditional on joint wasting and stunting exposures are then used to inform underweight exposures in our main simulation as described in the main text.

Notably, we also utilize this simulated population of 150,000 with correlated wasting, stunting, and underweight exposures to calculate custom joint population attributable fractions (PAFs) due to child growth failure (inclusive of wasting, stunting, and underweight risks) that reflect the specific correlation structure between wasting, stunting, and underweight modeled in our simulation rather than rely on PAFs estimated from the GBD 2021 study that do not account for this correlation. We do this by calculating a simulant-specific joint child growth failure relative risk value for a given outcome (e.g., diarrheal diseases incidence) equal to the product of the stunting, wasting, and underweight relative risk values corresponding to its stunting, wasting, and underweight exposures as informed from the GBD 2021 study. The joint CGF PAF for that outcome is then calculated as $\frac{\bar{RR}-1}{\bar{RR}}$, where $RR$ represents the mean joint CGF relative risk among the entire simulated population.

Calibration of wasting transition model

As described in the main text, transition rates used in the wasting transition model in our simulation are calibrated to a steady state equilibrium model, shown in the figure below. Our calibration model does not consider subcategories of moderate wasting, and we assume that each moderate wasting subcategory is subject to the same transition rates as the overall moderate wasting state in the calibration model.


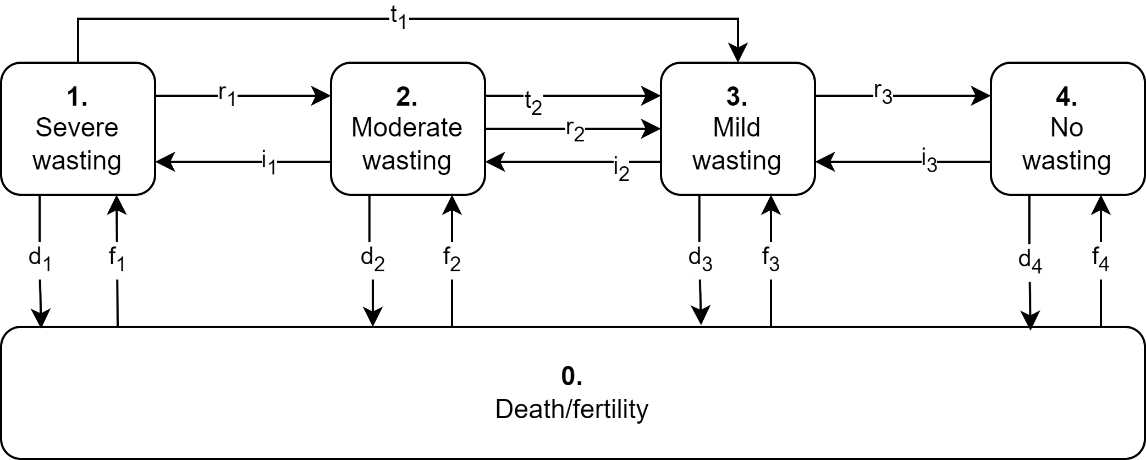


This steady state equilibrium model has several direct inputs, outlined below.

*Wasting prevalence*

GBD 2021 study estimates of four-category wasting exposure prevalence are used to inform the steady state exposure value for each category. Notably, these values are rescaled to include a fifth category that represented death, equal in prevalence to the all-cause mortality rate (ACMR) for the given age/sex/location demographic group.

Prevalence of each category *i* is represented as $p_{i}$

*Wasting mortality*

Estimates of four-category wasting exposure-specific mortality rates, derived from the GBD 2021 study, inform the transition rates from each wasting state to the death state. Wasting state-specific mortality rates are informed from (a) GBD 2021 estimates of location-, sex-, age-, and year-specific all-cause mortality rate values, (b) joint CGF PAFs calculated to reflect the modeled correlation between wasting, stunting, and underweight exposures as described in the previous section, (c) CGF risk effects as informed from the GBD 2021 study, and (d) mortality due to protein-energy malnutrition, which was proportionately distributed across moderate and severe wasting states.

Notably, in addition to transitions from each wasting state into the death state (*d* transitions), we also include transitions from the death state back to each wasting state (*f* transitions) in order to maintain steady state in our calibration model (this could also be considered “aging in” to the system). These transition rates are scaled to the prevalence of each wasting category. In other words, for wasting category *i*, $f_{i}= p_{i}$*.*

*Treated wasting recovery rates*

The transition rate for successfully treated moderate wasting recovery to mild wasting ($t_{2}$) is informed from the mean time to recovery of moderate acute malnutrition from the treatment arm of the ComPAS trial (Bailey et al., 2020) equal to 73 days. Notably, we assume that those unsuccessfully treated for moderate wasting recover according to the untreated recovery transition ($r_{2}$).

Transition rate for successfully treated severe wasting recovery to mild wasting ($t_{1}$) are informed from the mean time to recovery of severe acute malnutrition from the treatment arm of the ComPAS trial (Bailey et al., 2020) equal to 112 days. Notably, we assume that those unsuccessfully treated for severe wasting recover according to the untreated recovery transition ($r_{1}$).

*Wasting treatment rates*

Estimates of the baseline coverage of treatment of moderate wasting and severe wasting as well as estimates of the proportion of moderate and severe wasting cases covered by treatment interventions who are successfully treated to recovery were used to calibrate the proportion of the population transitioning through treated and untreated recovery rates from moderate and severe wasting.

| **Parameter** | **Definition** | **Value (95% UI)** | **Source** |
| --- | --- | --- | --- |
| $C_{1}$ | Proportion of severe wasting cases that receive treatment | 48.8% (37.4, 60.4) | (Isanaka et al., 2021) |
| $E_{1}$ | Proportion of treated cases of severe wasting that successfully respond to treatment | 70% (64, 76) | (Bitew et al., 2020) |
| $C_{2}$ | Proportion of moderate wasting cases that receive treatment | 15% (10, 20) | Assumption |
| $E_{2}$ | Proportion of treated cases of moderate wasting that successfully respond to treatment | 70% (64, 76) | Assumed to be the same as $E_{1}$ |

*“Incident” wasting transition rates*

Age-specific transition rate values from less severe to more severe wasting states (*i* transitions) were informed from a collection of 22 longitudinal cohorts from 11 LMICs in South Asia, Sub-Saharan Africa, and Latin America in which data were collected between 1985 and 2021. These cohorts were assembled using the Bill & Melinda Gates Foundation’s Knowledge Integration (ki) initiative database. We excluded studies that had concerns over generalizability of wasting transition rates due to study population and/or the presence of interventions that may directly impact transition rates. A list of included and excluded studies and their reasons for exclusion is included below. We analyzed transition rates among age categories of 1–5 months, 6–11 months, and 1–4 years.

- Included studies: CMC-V-BCS, CMIN, GMS_NEPAL, MAL-ED, NIH CRYPTO, PROVIDE, SAS VIT A, SAS VIT B12, SAS_COMPFEED, TANZANIA CHILD 2, UCDNUT_BFA_ZN, Zvitambo
- Excluded studies:
  - AKU_EE: excluded due to study population of infants with insufficient respond to ready to use therapeutic food
  - DIVIDS: excluded due to study population of small for gestational age infants
  - Ilins-Dose: excluded due to lipid-based nutrient supplementation intervention
  - Ilins-Dyad: excluded due to lipid-based nutrient supplementation intervention
  - SAS_LBW: excluded due to study population of low birthweight infants

These inputs to the steady state model then allowed us to solve for the following transition rates:

- Transition rate from mild wasting to no wasting ($r_{3}$)
- Untreated transition rate from moderate wasting to mild wasting ($r_{2}$)
- Untreated transition rate from severe wasting to moderate wasting ($r_{1}$)

We followed the following process to do so:

1. At the sex-, age-, and draw-specific level, randomly sample a study from included longitudinal studies from the Ki database.
2. Randomly sample event count values for the numerator of each “incident” wasting transition (*i* transitions) under the assumption of a Poisson distribution of uncertainty and divide by person-time denominators corresponding to the sampled Ki study.
3. Calculate output transition rates according to steady state equilibrium assumptions.
   1. We assumed the following equations were true at steady state equilibrium. Note that all transition rates (labeled in diagram above) represent the number of transitions through that pathway divided by person time in the source state for that transition.
      1. ${f_{4}p_{0}+r}_{3}p_{3}=i_{3}p_{4}+d_{4}p_{4}$
      2. ${f_{3}p_{0}+i}_{3}p_{4}+r_{2}p_{2}\left( 1-C_{2}E_{2} \right)+t_{2}p_{2}C_{2}E_{2}+t_{1}p_{1}C_{1}E_{1}=r_{3}p_{3}+i_{2}p_{3}+d_{3}p_{3}$
      3. $f_{2}p_{0}+i_{2}p_{3}+r_{1}p_{1}\left( 1-C_{1}E_{1} \right)=r_{2}p_{2}{(1-C}_{2}E_{2})+t_{2}p_{2}C_{2}E_{2}+i_{1}p_{2}+d_{2}p_{2}$
      4. $f_{1}p_{0}+i_{1}p_{2}=t_{1}p_{1}C_{1}E_{1}+r_{1}p_{1}\left( 1-C_{1}E_{1} \right)+d_{1}p_{1}$
   2. This allowed us to calculate values for the unknown parameters ${(r}_{3}, r_{2}, r_{1}$) according to the following equations. Note that there are two options for finding the value of $r_{2}$ that can be derived from the above equations, one based on maintaining equilibrium in the mild wasting state, and the other in the moderate wasting state. The two alternative values for parameter $r_{2}$ (equation ii and iii) were averaged for use as the final $r_{2}$ parameter value.
      1. $r_{3}=\frac{d_{4}p_{4}+i_{3}p_{4}-f_{4}p_{0}}{p_{3}}$
      2. $r_{2}=\frac{d_{3}p_{3}+i_{2}p_{3}+r_{3}p_{3}-t_{1}p_{1}C_{1}E_{1}-t_{2}p_{2}C_{2}E_{2}-i_{3}p_{4}-f_{3}p_{0}}{p_{2}\left( 1-C_{2}E_{2} \right)}$
      3. $r_{2}=\frac{f_{2}p_{0}+i_{2}p_{3}+r_{1}p_{1}\left( 1-C_{1}E_{1} \right)-t_{2}p_{2}C_{2}E_{2}-i_{1}p_{2}-d_{2}p_{2}}{p_{2}\left( 1-C_{2}E_{2} \right)}$
      4. $r_{1}=\frac{i_{1}p_{2}+f_{1}p_{0}-t_{1}p_{1}C_{1}E_{1}-d_{1}p_{1}}{p_{1}(1-C_{1}E_{1})}$
4. Assess validity of outputs based on the following criteria:
   1. All transition rates must be positive.
   2. Treated recovery rate of severe wasting to mild wasting ($t_{1}$) must be greater than the untreated recovery rate of severe wasting to moderate wasting ($r_{1}$).
   3. Treated recovery rate of moderate wasting to mild wasting ($t_{2}$) must be greater than the untreated recovery rate of moderate wasting to mild wasting ($r_{2}$).
   4. The two alternative values for$r_{2}$ (equation ii and equation iii in step 3b above) must be within 10% of one another.
5. If any of the validity criteria in the previous step are not satisfied, repeat from the first step until a valid result is obtained. Continue until 500 valid draws are generated for each age/sex/location group.

The resulting values for untreated recovery rates (*r* transitions), shown in transitions per person-year, are summarized in the table below along with their 95% uncertainty intervals (UIs), for the illustrative example model calibrated to Ethiopia as described in the main text of this paper.

| **Age group** | **Untreated transition rate from mild wasting to no wasting (**$\boldsymbol{r}_{\mathbf{3}}$**)** | **Untreated transition rate from moderate wasting to mild wasting (**$\boldsymbol{r}_{\mathbf{2}}$**)** | **Untreated transition rate from severe wasting to mild wasting (**$\boldsymbol{r}_{\mathbf{1}}$**)** |
| --- | --- | --- | --- |
| 1-5 months | 3.10 (0.81, 6.19) | 2.49 (0.41, 5.79) | 2.66 (0.08, 7.12) |
| 6-11 months | 4.19 (2.12, 6.48) | 3.05 (1.48, 4.31) | 1.17 (0.24, 2.30) |
| 12-23 months | 2.60 (0.98, 4.75) | 2.19 (1.13, 3.23) | 1.15 (0.21, 2.45) |
| 2-4 years | 2.88 (1.12, 5.27) | 2.47 (1.06, 3.79) | 1.13 (0.12, 2.41) |

Our approach is limited in at least four ways. First, we do not consider seasonal variation in wasting exposure or transition rates. Additionally, we do not consider individual heterogeneity in wasting transition rates and/or relapse dynamics following treatment. We rely on sparse data on wasting treatment and assume that child wasting as measured by WHZ is a reasonable proxy for acute malnutrition assessed by WHZ, mid-upper arm circumference (MUAC), and the presence of edema as is often typical for wasting treatment. Finally, we assume that those successfully treated for severe wasting transition directly to the mild wasting state without transitioning through the moderate wasting state. By definition, a transition through the moderate wasting state must occur in reality. However, this design was selected for convenient compatibility with the standard discharge criteria for severe wasting treatment used in studies that report treated severe wasting recovery rates.

Our approach benefits from using data on “incident” wasting transition rates paired with GBD estimates of child growth failure and literature on wasting treatment to inform transition rates of untreated moderate and severe wasting, which are generally unavailable in the literature as ethical constraints prevent the direct observation of the natural history of severe wasting without intervention. Notably, limited data on untreated recovery from moderate acute malnutrition do exist and largely support the findings of our calibration model (James et al. 2016).

## Appendix 5: Impact of birthweight on child growth failure

Stunting

We informed the effect of birthweight on child stunting exposure from evidence of an analysis of twin and sibling pairs in DHS data by (McGovern, 2019) that found the marginal effect of a 200-g increase in birthweight (at 2500 g) is associated with a 2.0 (SD: 0.6) to 2.3 (SD: 0.5) percentage point decrease in the probability of stunting among children under 5 years of age.

We did not model baseline correlation between birthweight and child stunting but did model changes in birthweight between simulated scenarios for individual simulants (due to changes in intervention exposures) resulting in associated changes in child stunting exposures. We did this by reducing the combined prevalence of moderate and severe categories of the population-level stunting exposure distribution used to benchmark individual-level stunting exposures by two percentage points (assuming proportionate relative decreases in the moderate and severe categories, respectively) and a two percentage-point increase in the mild stunting exposure category. This adjustment was applied to all age groups between 1 month and 5 years of age.

Wasting

We modeled an impact of birthweight on wasting exposure at 28 days of life such that infants classified as low birthweight (<2,500 grams at birth) were 1.82 (95% CI: 1.35, 2.45) times more likely to be wasted at 28 days of life than infants who were not classified as low birthweight (>2,500 grams at birth). We assumed that this relative risk applied equally to moderate and severe wasting categories and was associated with a proportionate increase in mild wasting and no wasting categories.

The magnitude of this effect was informed from an analysis of DHS data, in which we calculated the relative risk of birthweight less than 2,500 grams on the outcome of WHZ < -2 among those 2 months of age or less for the most recent round of available DHS round 7 and 8 and then meta-analyzed the results under the assumption of random effects (forest plot shown below). We found that there was no significant heterogeneity by geographic region, so we proceeded with the overall effect estimate pooled across all analyzed locations.

Beyond the effect of birthweight on wasting state at 28 days, we assumed that there was no direct effect of birthweight exposure on wasting transition rates thereafter. This assumption is supported by evidence from (Mertens et al., 2023) that found gestational age at birth (which is highly correlated with birthweight) was not associated with wasting incidence from 0 to 6 months of age. Notably, this analysis found that preterm birth was positively associated with wasting incidence at 6–24 months of age; however, we hypothesize that this association may be driven by a common cause between these variables (such as food insecurity) rather than a causal impact, given that we would expect a causal impact to be apparent in the 0–6-month age group as well if it existed.


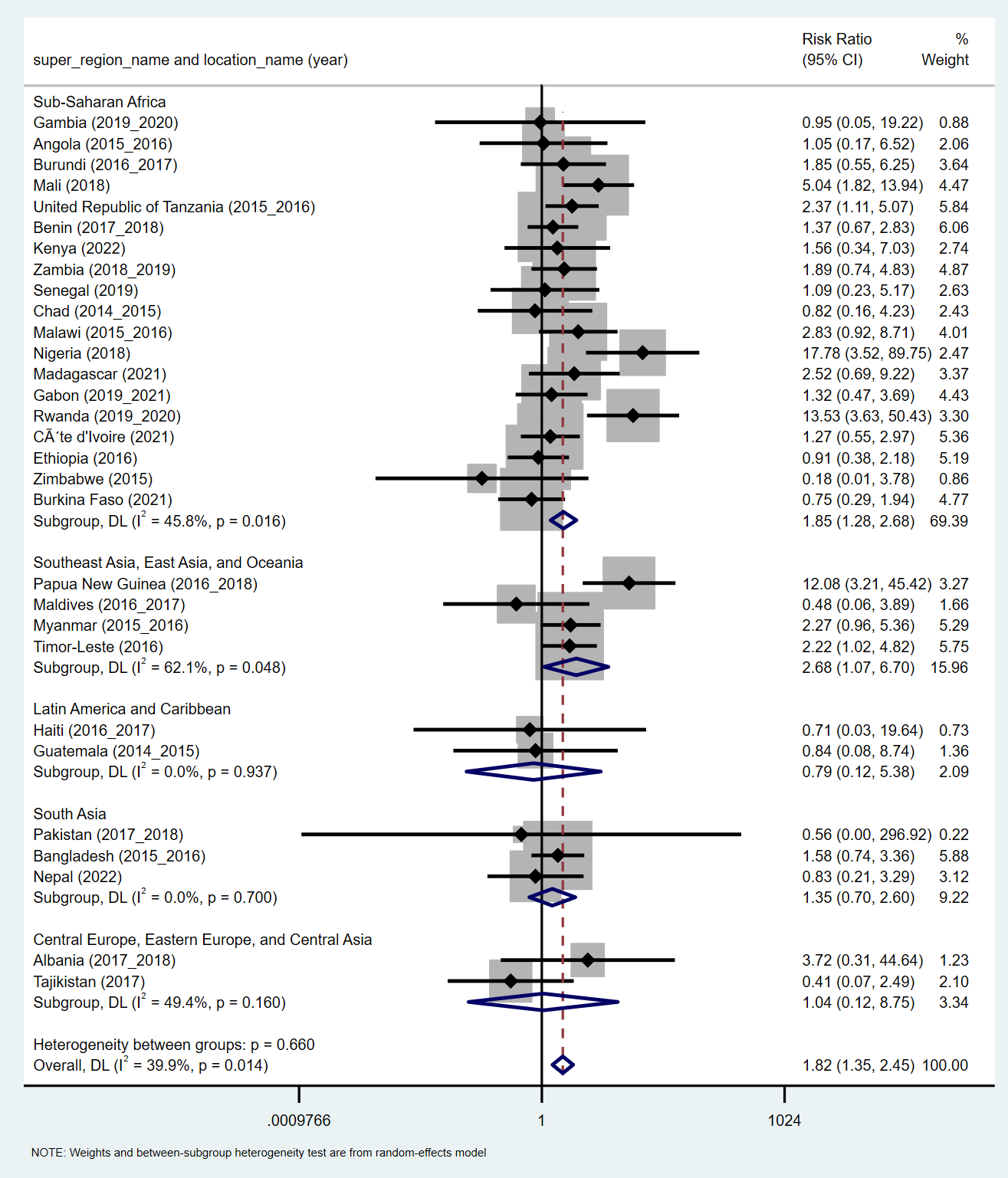


## Appendix 6: Small-quantity lipid-based nutrient supplementation intervention effects

Stunting

The impact of SQ-LNS on stunting exposure in our model was informed from an individual participant (IPD) data meta-analysis performed by (Dewey et al., 2021). While effects specific to severe stunting were available in (Dewey et al., 2022), the study authors provided us with additional unpublished effect estimates on moderate, mild, and non-stunted categories as well. The prevalence ratio for mild stunting did not significantly differ from 1, so we assumed that the prevalence ratio for this category was equal to 1. Additionally, rather than utilize the prevalence ratio for the non-stunted category from these results, we modeled an increase in the non-stunted exposure category equal in magnitude to the decreases in the moderate and severe stunting exposure categories. In our model, we modified the population-level stunting exposure used to benchmark individual-level stunting exposures in accordance with the prevalence ratios as described above for those covered by the SQ-LNS supplementation intervention from the beginning of supplementation at 6 months of age through 5 years of age.

Wasting

As for stunting, we also informed the effect of the SQ-LNS intervention on wasting from data provided from the IPD meta-analysis performed by (Dewey et al., 2021) study authors. Notably, these data were provided as wasting prevalence ratios; however, as we utilized a transition rather than prevalence-based model of wasting in our simulation, we could not directly apply these prevalence ratios in our model.

Therefore, we calibrated an individual-based microsimulation of wasting transition rates to estimate effects of the SQ-LNS intervention on wasting transition rates that resulted in the prevalence ratios observed in the IPD meta-analysis. Notably, the SQ-LNS intervention may impact wasting prevalence through reductions in wasting incidence, increases in wasting recovery, or some combination of the two. Few studies have directly measured impacts on wasting transition rates; however, limited evidence from (Huybregts et al., 2019) suggests that the intervention may influence wasting prevalence primarily through reductions in wasting incidence (with a relative risk value on all acute malnutrition episodes of 0.69) rather than increases in wasting recovery rates (with no significant difference found in the length of treatment between treatment arms). Therefore, we made the simplifying assumption in our model that the SQ-LNS intervention had no impact on wasting recovery rates.

Due to the finding by (Huybregts et al., 2019) that “the difference between study arms in the probability of developing the first [acute malnutrition] episode mainly occurred during the first 4 months of follow-up and then remained constant” (p. 19), we implemented age-specific effects such the prevalence ratios from the meta-analysis are achieved at 10 months of age (four months following the initiation of supplementation at six month of age) and maintained through 18 months of age (12 months supplementation duration). Results from the calibration model were applied in our main simulation and are summarized in the table below. Notably, these values were calibrated to the child population in Ethiopia and did not include mortality; the calibration may not hold for all other populations.

| **Wasting transition** | **Relative risk (95% uncertainty interval) among 6–10-month age group** | **Relative risk (95% uncertainty interval) among 10–18-month age group** |
| --- | --- | --- |
| No wasting to mild wasting | 0.81 (0.73, 0.93) | 0.90 (0.85, 0.97) |
| Mild wasting to moderate wasting | 0.69 (0.55, 0.89) | 0.90 (0.84, 0.97) |
| Moderate wasting to severe wasting | 0.27 (0.05, 0.67) | 0.79 (0.68, 0.90) |

Other outcomes

We did not model impacts of the SQ-LNS intervention on anemia, vitamin A deficiency, or developmental outcomes despite evidence that it may affect these outcomes (Prado et al., 2021; Wessells et al., 2021). Additionally, we did not model any direct impact of the SQ-LNS intervention on child mortality outside of the pathway mediated through its impacts on wasting and stunting and their associated effects on mortality in our simulation model, which may differ from the reported effects of the intervention on child mortality in (Stewart et al., 2019).

## Appendix 7: Scenario layout, optimization function and constraints

Scenario layout

We ran the health model for every possible combination of our modeled interventions. For instance, if we only considered IFA and SAM treatment interventions, we would run the health model under the following conditions:

0% coverage IFA, 0% coverage SAM treatment

100% coverage IFA, 0% coverage SAM treatment

0% coverage IFA, 100% coverage SAM treatment

100% coverage IFA, 100% coverage SAM treatment

We rely on the key assumption that individuals within our simulated population are independent of one another in that the health status of one individual does not impact any others. This allows us to assume that the population health status under 50% coverage of a given intervention can be equivalently represented as the average between the population health status under 100% coverage of the intervention and the population health status under 0% coverage.

With all seven of our modeled interventions, after excluding illogical combinations (such as simultaneous presence of universal MAM treatment and targeted MAM treatment interventions), this amounts to a total of 48 scenarios in addition to one baseline scenario. In each of these scenarios, we record counts of deaths, stillbirths, YLLs, YLDs, incident wasting cases, person-time (total time simulants spent alive), and intervention administration counts that occurred in our simulated populations. Deaths, YLLs, and YLDs are stratified by pregnancy and child populations, and person-time counts are stratified by stunting state and age group for children.

Across modeled scenarios, we utilize common random numbers (Flaxman et al., 2017) at the individual simulant level such that in the absence of intervention influence, each simulant would undergo the exact same trajectory in one scenario as the others, reducing noise across scenarios in our model.

Optimization function and constraints

We use the scipy.optimize package in Python (Virtanen et al., 2020) to perform our allocative efficiency analysis. Inputs to our optimization function include the recorded health outcomes for each modeled scenario and the calculated cost of each scenario (obtained by multiplying the recorded intervention administration counts for each scenario by the intervention unit costs). The optimization objective is to find the fractional combination of scenarios that maximizes/minimizes the specified health quantity (ex: minimize DALYs or maximize person-time) under the following constraints:

Total cost cannot exceed user-defined budget envelope

Total intervention-specific coverage cannot exceed specified saturation coverage limits

The two intervention coverage rules described below are followed

Intervention coverage rule one: all interventions share a “common care-seeking propensity” that dictates if population coverage of intervention A and B are both 50%, we assume the same 50% of the population is covered by interventions A and B and that the remaining 50% of the population has access to neither intervention A nor B.

Intervention coverage rule two: we assume that ANC intervention products could not be “targeted” to a subpopulation based on their coverage of child nutrition interventions or vice versa. For instance, if saturation coverage of ANC products is greater than that of child nutrition interventions, a solution that allocates IFA to the portion of the population with access to child nutrition interventions and MMN to the portion of the population without access to child nutrition interventions is not allowed. This is because it would require knowing a future event (child intervention coverage) at the time of pregnancy, which is not possible.

Following these and the previously stated assumption of simulant independence, we calculate overall cost, health impact, and intervention coverage as outputs of our optimization function for a single budget size.

## Appendix 8: Monte Carlo draws and propagating uncertainty

Monte Carlo draws

In order to account for parameter uncertainty in the model, we utilized different Monte Carlo uncertainty draws for all input values. The GBD study generates 500 draws for each parameter estimate they produce. These are basically 500 different possible values for each parameter that are estimated based on the underlying data sources. They are designed to acknowledge the inherent uncertainty in estimating parameters. For inputs that were not from GBD, we generated 500 draws based on the mean and uncertainty interval in the relevant data source. To run the model, we then randomly selected a subset of the uncertainty draws and used these as the input values to the model. For the pregnancy simulation, we used 100 draws, and 20 of these were randomly for use in the child simulation.

Propagating uncertainty

We ran the model separately for each draw selected. Results can therefore be generated at the draw level, as well as averaging across draws. By finding draw-level results, we can assess how sensitive the model is to input parameters. If there is significant variation in final results across draws, it indicates that the model results are highly sensitive to small changes in input parameters, while if all draw-level results provide the same answer, it indicates that the model is not sensitive to small changes.

# References

Aguree, S., & Gernand, A. D. (2019). Plasma volume expansion across healthy pregnancy: A systematic review and meta-analysis of longitudinal studies. *BMC Pregnancy and Childbirth*, *19*(1), 508. https://doi.org/10.1186/s12884-019-2619-6

Bailey, J., Opondo, C., Lelijveld, N., Marron, B., Onyo, P., Musyoki, E. N., Adongo, S. W., Manary, M., Briend, A., & Kerac, M. (2020). A simplified, combined protocol versus standard treatment for acute malnutrition in children 6–59 months (ComPAS trial): A cluster-randomized controlled non-inferiority trial in Kenya and South Sudan. *PLOS Medicine*, *17*(7), e1003192. https://doi.org/10.1371/journal.pmed.1003192

Bitew, Z. W., Alemu, A., & Worku, T. (2020). Treatment outcomes of severe acute malnutrition and predictors of recovery in under-five children treated within outpatient therapeutic programs in Ethiopia: A systematic review and meta-analysis. *BMC Pediatrics*, *20*(1), 335. https://doi.org/10.1186/s12887-020-02188-5

Brauer, M., Roth, G. A., Aravkin, A. Y., Zheng, P., Abate, K. H., Abate, Y. H., Abbafati, C., Abbasgholizadeh, R., Abbasi, M. A., Abbasian, M., Abbasifard, M., Abbasi-Kangevari, M., ElHafeez, S. A., Abd-Elsalam, S., Abdi, P., Abdollahi, M., Abdoun, M., Abdulah, D. M., Abdullahi, A., … Gakidou, E. (2024). Global burden and strength of evidence for 88 risk factors in 204 countries and 811 subnational locations, 1990–2021: A systematic analysis for the Global Burden of Disease Study 2021. *The Lancet*, *403*(10440), 2162–2203. https://doi.org/10.1016/S0140-6736(24)00933-4

Dewey, K. G., Arnold, C. D., Wessells, K. R., Prado, E. L., Abbeddou, S., Adu-Afarwuah, S., Ali, H., Arnold, B. F., Ashorn, P., Ashorn, U., Ashraf, S., Becquey, E., Brown, K. H., Christian, P., Colford, Jr, J. M., Dulience, S. J., Fernald, L. C., Galasso, E., Hallamaa, L., … Stewart, C. P. (2022). Preventive small-quantity lipid-based nutrient supplements reduce severe wasting and severe stunting among young children: An individual participant data meta-analysis of randomized controlled trials. *The American Journal of Clinical Nutrition*, *116*(5), 1314–1333. https://doi.org/10.1093/ajcn/nqac232

Dewey, K. G., Stewart, C. P., Wessells, K. R., Prado, E. L., & Arnold, C. D. (2021). Small-quantity lipid-based nutrient supplements for the prevention of child malnutrition and promotion of healthy development: Overview of individual participant data meta-analysis and programmatic implications. *The American Journal of Clinical Nutrition*, *114*, 3S-14S. https://doi.org/10.1093/ajcn/nqab279

Flaxman, A. D., Deason, A. W., Dolgert, A. J., Mumford, J. E., Sorensen, R. J. D., Eldrenkamp, E., Vos, T., Foreman, K., Mokdad, A. H., & Weaver, M. R. (2017). Untangling uncertainty with common random numbers: A simulation study. *Summer Simulation Multi-Conference*, *31*, 1–12.

Hambidge, K. M., Westcott, J. E., Garcés, A., Figueroa, L., Goudar, S. S., Dhaded, S. M., Pasha, O., Ali, S. A., Tshefu, A., Lokangaka, A., Derman, R. J., Goldenberg, R. L., Bose, C. L., Bauserman, M., Koso-Thomas, M., Thorsten, V. R., Sridhar, A., Stolka, K., Das, A., … Krebs, N. F. (2019). A multicountry randomized controlled trial of comprehensive maternal nutrition supplementation initiated before conception: The Women First trial. *The American Journal of Clinical Nutrition*, *109*(2), 457–469. https://doi.org/10.1093/ajcn/nqy228

Huybregts, L., Le Port, A., Becquey, E., Zongrone, A., Barba, F. M., Rawat, R., Leroy, J. L., & Ruel, M. T. (2019). Impact on child acute malnutrition of integrating small-quantity lipid-based nutrient supplements into community-level screening for acute malnutrition: A cluster-randomized controlled trial in Mali. *PLOS Medicine*, *16*(8), e1002892. https://doi.org/10.1371/journal.pmed.1002892

Isanaka, S., Andersen, C. T., Cousens, S., Myatt, M., Briend, A., Krasevec, J., Hayashi, C., Mayberry, A., Mwirigi, L., & Guerrero, S. (2021). Improving estimates of the burden of severe wasting: Analysis of secondary prevalence and incidence data from 352 sites. *BMJ Global Health*, *6*(3), e004342. https://doi.org/10.1136/bmjgh-2020-004342

McGovern, M. E. (2019). How much does birth weight matter for child health in developing countries? Estimates from siblings and twins. *Health Economics*, *28*(1), 3–22. https://doi.org/10.1002/hec.3823

Mertens, A., Benjamin-Chung, J., Colford, J. M., Coyle, J., Van Der Laan, M. J., Hubbard, A. E., Rosete, S., Malenica, I., Hejazi, N., Sofrygin, O., Cai, W., Li, H., Nguyen, A., Pokpongkiat, N. N., Djajadi, S., Seth, A., Jung, E., Chung, E. O., Jilek, W., … Yori, P. P. (2023). Causes and consequences of child growth faltering in low-resource settings. *Nature*, *621*(7979), 568–576. https://doi.org/10.1038/s41586-023-06501-x

Naghavi, M., Ong, K. L., Aali, A., Ababneh, H. S., Abate, Y. H., Abbafati, C., Abbasgholizadeh, R., Abbasian, M., Abbasi-Kangevari, M., Abbastabar, H., Abd ElHafeez, S., Abdelmasseh, M., Abd-Elsalam, S., Abdelwahab, A., Abdollahi, M., Abdollahifar, M.-A., Abdoun, M., Abdulah, D. M., Abdullahi, A., … Murray, C. J. L. (2024). Global burden of 288 causes of death and life expectancy decomposition in 204 countries and territories and 811 subnational locations, 1990–2021: A systematic analysis for the Global Burden of Disease Study 2021. *The Lancet*, S0140673624003672. https://doi.org/10.1016/S0140-6736(24)00367-2

Omotayo, M. O., Abioye, A. I., Kuyebi, M., & Eke, A. C. (2021). Prenatal anemia and postpartum hemorrhage risk: A systematic review and meta‐analysis. *Journal of Obstetrics and Gynaecology Research*, *47*(8), 2565–2576. https://doi.org/10.1111/jog.14834

Prado, E. L., Arnold, C. D., Wessells, K. R., Stewart, C. P., Abbeddou, S., Adu-Afarwuah, S., Arnold, B. F., Ashorn, U., Ashorn, P., Becquey, E., Brown, K. H., Chandna, J., Christian, P., Dentz, H. N., Dulience, S. J. L., Fernald, L. C. H., Galasso, E., Hallamaa, L., Hess, S. Y., … Dewey, K. G. (2021). Small-quantity lipid-based nutrient supplements for children age 6–24 months: A systematic review and individual participant data meta-analysis of effects on developmental outcomes and effect modifiers. *The American Journal of Clinical Nutrition*, *114*(Supplement_1), 43S-67S. https://doi.org/10.1093/ajcn/nqab277

Sharma, R., & Sharma, S. (2023). *Physiology, Blood Volume*. StatPearls Publishing. https://www.ncbi.nlm.nih.gov/books/NBK526077/

Stewart, C. P., Wessells, K. R., Arnold, C. D., Huybregts, L., Ashorn, P., Becquey, E., Humphrey, J. H., & Dewey, K. G. (2019). Lipid-based nutrient supplements and all-cause mortality in children 6–24 months of age: A meta-analysis of randomized controlled trials. *The American Journal of Clinical Nutrition*, nqz262. https://doi.org/10.1093/ajcn/nqz262

Virtanen, P., Gommers, R., Oliphant, T. E., Haberland, M., Reddy, T., Cournapeau, D., Burovski, E., Peterson, P., Weckesser, W., Bright, J., Van Der Walt, S. J., Brett, M., Wilson, J., Millman, K. J., Mayorov, N., Nelson, A. R. J., Jones, E., Kern, R., Larson, E., … Vázquez-Baeza, Y. (2020). SciPy 1.0: Fundamental algorithms for scientific computing in Python. *Nature Methods*, *17*(3), 261–272. https://doi.org/10.1038/s41592-019-0686-2

Wessells, K. R., Arnold, C. D., Stewart, C. P., Prado, E. L., Abbeddou, S., Adu-Afarwuah, S., Arnold, B. F., Ashorn, P., Ashorn, U., Becquey, E., Brown, K. H., Byrd, K. A., Campbell, R. K., Christian, P., Fernald, L. C., Fan, Y.-M., Galasso, E., Hess, S. Y., Huybregts, L., … Dewey, K. G. (2021). Characteristics that modify the effect of small-quantity lipid-based nutrient supplementation on child anemia and micronutrient status: An individual participant data meta-analysis of randomized controlled trials. *The American Journal of Clinical Nutrition*, *114*, 68S-94S. https://doi.org/10.1093/ajcn/nqab276
